# Bidirectional sequestration between a bacterial hibernation factor and a glutamate metabolizing protein

**DOI:** 10.1073/pnas.2207257119

**Published:** 2022-09-19

**Authors:** David Ranava, Christopher M. Scheidler, Martin Pfanzelt, Michaela Fiedler, Stephan A. Sieber, Sabine Schneider, Mee-Ngan F. Yap

**Affiliations:** ^a^Department of Microbiology-Immunology, Northwestern University Feinberg School of Medicine, Chicago, IL 60611, USA;; ^b^Department of Chemistry, Ludwig-Maximilians-Universität München, 81377 Munich, Germany;; ^c^Department of Chemistry, Chair of Organic Chemistry III, Center for Functional Protein Assemblies (CPA), Technische Universität München, 80333 Garching, Germany

**Keywords:** ribosome, glutamate dehydrogenase, *Staphylococcus aureus*, hibernation, moonlighting protein

## Abstract

Bacterial hibernating 100S ribosomes (the 70S dimers) are excluded from translation and are protected from ribonucleolytic degradation, thereby promoting long-term viability and increased regrowth. No extraribosomal target of any hibernation factor has been reported. Here, we discovered a previously unrecognized binding partner (YwlG) of hibernation-promoting factor (HPF) in the human pathogen *Staphylococcus aureus*. YwlG is an uncharacterized virulence factor in *S. aureus*. We show that the HPF–YwlG interaction is direct, independent of ribosome binding, and functionally linked to cold adaptation and glucose metabolism. Consistent with the distant resemblance of YwlG to the hexameric structures of nicotinamide adenine dinucleotide (NAD)–specific glutamate dehydrogenases (GDHs), YwlG overexpression can compensate for a loss of cellular GDH activity. The reduced abundance of 100S complexes and the suppression of YwlG-dependent GDH activity provide evidence for a two-way sequestration between YwlG and HPF. These findings reveal an unexpected layer of regulation linking the biogenesis of 100S ribosomes to glutamate metabolism.

## Significance

Ribosome hibernation is a conserved mechanism used by both bacteria and eukaryotes to occlude translation and to extend organismal lifespan. Here, we describe a dual-function, hibernation factor hibernation-promoting factor (HPF) in pathogenic *Staphylococcus aureus* that dimerizes 70S ribosomes and represses YwlG-mediated glutamate dehydrogenase activity via a physical HPF–YwlG interaction. Conversely, YwlG binding to HPF reduces the formation of 100S ribosomes. Our data support the genetic and functional links between HPF and YwlG in the cold response and glucose tolerance. Both HPF and YwlG are validated virulence factors, and our data pave the way to further disentangle the interconnection between bifunctional HPF and YwlG and their multimer assembly.

**Fig. 1.**
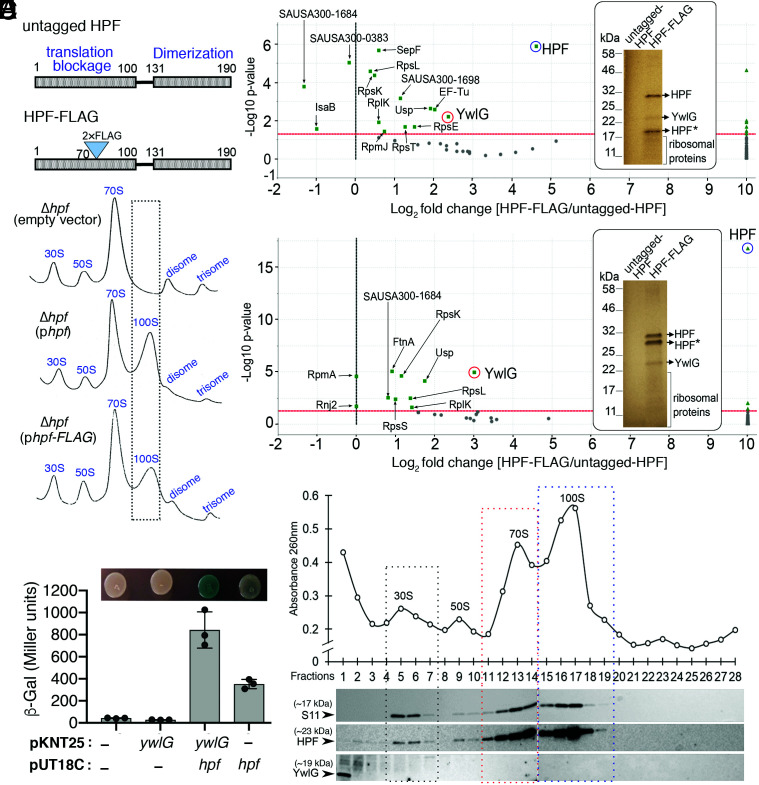
Identification of HPF interaction partners in *S. aureus*. (*A*) Schematic diagram showing the domain structures of *S. aureus* HPF and the position of the 2×FLAG in-frame fusion. The N-terminal domain (NTD) of HPF prevents translational initiation, whereas the carboxyl-terminal domain (CTD) promotes the dimerization of 70S ribosomes to form the 100S complex. (*B*) Ribosome sedimentation profiles of the Δ*hpf*-null mutant bearing either empty vector, untagged HPF, or 2×FLAG-HPF. Five Abs_260_ units of RNA were loaded on a 5–30% sucrose gradient (x axes), and the levels of ribosomal complexes were monitored by the Abs_260_ (y axes). FLAG-tag insertion in HPF did not affect ribosome assembly and only modestly reduced the formation of 100S ribosomes. (*C* and *D*) Affinity copurification of HPF-binding proteins. TSB cultures of strains from *A* were harvested during the exponential (*C*) (4 h) or stationary phase (*D*) (18 h) at 37 °C. The total proteins were incubated with anti-FLAG M2 magnetic beads, and HPF and HPF-binding candidates were eluted as described in the Materials and Methods. Ten percent of the eluate was analyzed on an SDS-PAGE gel (*Insets*, images were pseudocolored), and protein bands that were unique in FLAG-tagged samples were identified by trypsin digestion followed by liquid chromatography (LC)-MS/MS. HPF* indicates degraded intermediates. The remaining eluate was directly subjected to gel-free quantitative MS, and differentially enriched proteins were visualized in Scaffold 4.11.0 and are presented in volcano plots (*n* = 2, *P* < 0.005, Fisher’s test with Benjamini–Hochberg parameters). (*E*) Validation of the HPF–YwlG interaction using a bacterial 2-hybrid (BACTH) system. T25-YwlG and T18-HPF chimeras are indicated on the x axis. High *β*-galactosidase activity is indicative of a direct YwlG–HPF interaction, consistent with chromogenic detection of LacZ accumulation on LB-X-Gal-isopropyl ß-D-1-thiogalactopyranoside solid agar after 24–30 h at 30 °C (*Top*). Each bar represents the average of three independent biological replicates (±SD). (*F*) YwlG does not associate with the mature ribosomes. Ribosome sedimentation profile of the wild-type JE2 strain grown in TSB to late exponential phase (optical density at 600 nm [OD_600_] = 1.6) at 37 °C. Five Abs_260_ units of RNA were ultracentrifuged through a 5–30% sucrose gradient (x axis), and the abundance of ribosomal complexes was monitored by Abs_260_ (y axis). Fractions were collected and probed with anti-HPF, anti-YwlG, and anti-S11 (30S subunit marker) on Western blots.

The dimerization of 70S ribosomes to form the translationally inactive hibernating 100S complex is a universal strategy employed by most, if not all, bacteria to maintain long-term viability and exit from quiescence (1, 2). Ribosome hibernation is also widespread among eukaryotes (see review (3)). While γ-proteobacteria (e.g., *Escherichia coli* and *Pseudomonas aeruginosa*) utilize both ribosome modulation factor (RMF) and hibernation-promoting factor (HPF) to induce 70S dimerization, the majority of bacteria use a single long form of HPF to promote the formation of the 100S complex. In the opportunistic human pathogen *Staphylococcus aureus*, the 190 amino acid–long HPF consists of a self-dimerizing carboxyl-terminal domain (CTD) and a translation-blocking N-terminal domain (NTD) connected by a 30-amino-acid (a.a.) unstructured linker (Fig. 1*A*). Cryoelectron microscopy structures of the *S. aureus* 100S ribosomes have shown that the NTD-HPF sterically occludes mRNA entry and transfer RNA binding to the A site and P site of the 30S decoding center, resulting in translational avoidance. The CTD-HPF at the interface of 30S-30S on one 70S directly interacts with another CTD-HPF that is tethered to the opposite copy of the 70S, resulting in “side-by-side” conjoining of the two 70S monomers (4, 5). In contrast, the *E. coli* 70S dimer adopts a “back-to-back” joining conformation (6, 7). Although *S. aureus* HPF forms a homodimer in vitro and binds both individual 30S subunits and 70S complexes (5), 30S-30S dimers have not been observed in vivo.

Despite the variation in hibernation factors and distinct dimeric architecture of the 100S complexes (5–11), the loss of HPF or RMF homologs leads to one or more phenotypes, including decreased long-term viability and regrowth (12–14), reduced antibiotic and stress tolerance (8, 15–17), and accelerated ribosome degradation and damage (12, 13, 18–23). Recent studies have shown that *S. aureus* HPF binding protects the 30S subunit and 70S ribosome from degradation by the 3′–5′ exoribonuclease (RNase) R and an unidentified RNase at three functionally important regions of 16S ribosomal RNA (rRNA) (24), whereas in *E. coli* lacking RMF, HPF, and YfiA, 16S rRNA is degraded by the concerted action of RNase R and the endonuclease YbeY at sites that are distinct from those found in *S. aureus* (25). The rapid ribosome turnover observed in an *S. aureus* Δ*hpf*-null mutant is linked to ∼1,000-fold and ∼100-fold reductions in murine infections and viability in long-term cultures in vitro, respectively (13, 26). Intriguingly, *S. aureus* constitutively expresses *hpf* throughout the cell growth cycle (13, 27–29), overexpression of HPF does not significantly retard cell growth (13), and global translation is only modestly compromised in HPF-proficient cells (13). These observations have led to an emerging hypothesis that the primary role of HPF is to prevent ribosome degradation rather than translational inhibition (as previously envisioned) and that translational avoidance is a consequence of HPF-NTD occupancy at the 30S decoding center.

The reversal of ribosome hibernation via the disassembly of 100S complexes is thought to promote bacterial regrowth and long-term survival, during which the dissociated 30S, 50S, and 70S ribosomes are presumably recycled for translational restart. In *S. aureus*, the dissociation involves the major ribosome recycling factor/elongation factor G (EF-G) pair and an alternative guanosine triphosphatase (GTPase) HflX that split the 100S ribosomes in a GTP hydrolysis–dependent manner under nonstressed conditions and heat stress, respectively (26, 30, 31). This mechanism suggests that assembly and disassembly of 100S ribosomes are dynamic and partially explains the lack of translational derepression in a Δ*hpf*-null mutant (13). *S. aureus* HPF is a highly abundant protein, so how the cells avoid rebinding of HPF to ribosomes following 100S complex disassembly is unclear. One possibility is that mRNAs may compete for the 30S subunits during translational initiation. This model has not been validated directly and cannot be applied to 70S ribosomes, which are the major targets of HPF. Rapid HPF turnover is unlikely, as HPF remains abundant even when no mature ribosomes are detected (13). Alternatively, the HPF–ribosome interaction may be interposed by a previously unrecognized factor that titrates HPF away from ribosomes. Motivated by this latter hypothesis, we identified an uncharacterized virulence factor, YwlG, that directly interacts with HPF and reduces the formation of 70S dimers in a methicillin-resistant *S. aureus* (MRSA). The HPF–YwlG interaction is independent of ribosome binding. Inactivation of *ywlG* suppresses the cold sensitivity and glucose susceptibility phenotypes of a Δ*hpf*-null mutant. We solved the X-ray crystallography structure of YwlG to 1.75 Å and found that HPF-CTD is critical for HPF–YwlG interactions. Unexpectedly, we found that YwlG is able to functionally compensate for the loss of a NAD-specific glutamate dehydrogenase GudB. The surprising physical and functional association of HPF and YwlG unveils an additional layer of regulation controlling the cellular concentrations of 100S ribosomes and key metabolic pathway.

## Results

### HPF Directly Interacts with a Cytoplasmic YwlG Independent of Ribosome Association.

To test the hypothesis that HPF is sequestered by a hitherto unknown factor, we constructed a FLAG-tagged HPF as a bait to identify potential HPF binding partners using a whole-cell lysate affinity purification approach and compared the enrichment of copurified proteins with that of an untagged HPF control. HPF is extremely sensitive to mutational perturbation in the NTD–CTD linker region and cannot tolerate affinity tagging at its *N* and carboxyl termini (8, 13). We found that an insertion of a tandem FLAG tag at the position 70 of the protein resulted in less perturbation of 70S dimerization than various affinity-tagged fusions (Fig. 1*A*). The untagged HPF and 2×FLAG HPF were expressed on a medium-copy plasmid under the control of the native *hpf* promoter. We confirmed that 2×FLAG HPF is able to restore the formation of 100S ribosomes in a Δ*hpf* knockout strain, albeit at a lower level than the untagged HPF (Fig. 1*B*). *S. aureus* Δ*hpf* cells carrying the HPF-expressing plasmids were harvested at the exponential growth phase (Fig. 1*C*) and stationary phase (Fig. 1*D*). Our label-free quantitative mass spectrometry analyses revealed that, aside from the expected HPF bait, ribosomal proteins (RpxX, x indicates any letters), a universal stress protein (Usp homolog, SAUSA300_1652), and a translation factor (EF-Tu) were copurified. Additionally, a protein of unknown function (YwlG, locus tag SAUSA300_2068) was enriched by 5-fold to 16-fold during the logarithmic growth and stationary phases, respectively. YwlG enrichment was also detectable on a sodium dodecyl sulfate polyacrylamide gel electrophoresis (SDS-PAGE) gel stained with EZ-Blue following mass spectrometric verification (Fig. 1 *C* and *D*, *Insets*). The results indicate that YwlG is a binding partner and potentially a sequestrator of HPF.

The HPF–YwlG association detected in the affinity pull-down assay could have been indirect. To rule out this possibility, we used the bacterial two-hybrid (BACTH) system to confirm a direct interaction between HPF and YwlG. The BACTH system is based on the interaction-mediated reconstruction of a cyclic adenosine monophosphate (cAMP) signaling cascade (32). YwlG and HPF are each genetically fused to either the T25 or T18 fragment of the adenylate cyclase (Cya) catalytic domain from *Bordetella pertussis* and coexpressed in an *E. coli* Δ*cya* strain. Functional complementation between T25 and T18 via a direct interaction of heterologous gene products leads to the production of cAMP, in turn activating the expression of a β-galactosidase LacZ reporter. We found that high β-galactosidase activity was only observed when both T25-YwlG and T18-HPF were coexpressed, not when either was expressed by itself, although T18-HPF alone produced basal LacZ levels. The quantitative β-galactosidase activity results were consistent with the colorimetric visualization of LacZ production (Fig. 1*E*). The BACTH results confirm that the Cya catalytic activity is mediated by the physical interactions between HPF and YwlG.

*S. aureus* HPF predominantly binds to 70S and 100S ribosomes and to a lesser extent to the 30S subunits. It is possible that ribosomes act as mediators of HPF–YwlG binding. To exclude this possibility, we performed sucrose density gradient ultracentrifugation to fractionate ribosomes into subunits and complexes. Each fraction was then collected to probe for the presence of HPF and YwlG by Western blotting. The quality of fractionation was monitored by the distribution of the 30S ribosomal protein S11. We first confirmed that the newly raised polyclonal YwlG antibody is specific (*SI Appendix*, Fig. S1). As expected, HPF and S11 were cofractionated with 30S, 70S, and 100S ribosomes, but YwlG was only found in the ribosome-free, low-density fractions (Fig. 1*F*), indicating that YwlG is not associated with mature ribosomes and does not bind to HPF-bound ribosomes. Collectively, the data from the unbiased affinity copurification, targeted BACTH, and the ribosome fractionation confirm that the *S. aureus* HPF–YwlG interaction is direct and specific, occurs extraribosomally, and can be recapitulated heterologously in *E. coli.*

### Distribution and Possible Roles of YwlG.

*S. aureus* YwlG is a 174-a.a.-long uncharacterized protein member of the UPF0340 family with a DUF436 domain of unknown function. A phylogenetic tree constructed for a selected set of 256 YwlG homologs from representative bacterial phyla revealed that YwlG homologs are almost exclusively found in the Firmicutes phylum, with a few exceptions found in the phyla of Deinococcota, Fibrobacteres, Bacteroidetes, and Spirochaeta (*SI Appendix*, Fig. S2). Incidentally, all bacterial species that carry YwlG homologs only harbor HPF homologs that have an extended carboxyl-terminal dimerization domain (Fig. 1*A*). Multiple sequence alignment of YwlG homologs revealed distinct regions of conservation (*SI Appendix*, Fig. S3). A previous chemical proteomic screen has suggested that *S. aureus* YwlG binds to pyridoxal phosphate (PLP) (33), a B6 vitamer that serves as the cofactor of many enzymes involved in a.a. transformation. However, YwlG–PLP binding has not been experimentally validated in vivo and the putative PLP binding residue (K58 in *S. aureus*) is not conserved among YwlG homologs (*SI Appendix*, Fig. S3). A Δ*ywlG* knockout did not exhibit growth defects in laboratory rich medium, changes in cell morphology, or sensitivity to oxidative, osmotic, and heat stress. However, a Δ*ywlG* mutant has been found to exhibit 50% compromised lung colonization in a model of acute murine pneumonia (34) and to exhibit reduced fitness during osteomyelitis (35), indicating that YwlG is critical for staphylococcal pathogenesis.

### Inactivation of *ywlG* Suppresses the Cold Sensitivity of Δ*hpf* Null Mutant in Lysogeny Broth (LB).

**Fig. 2.**
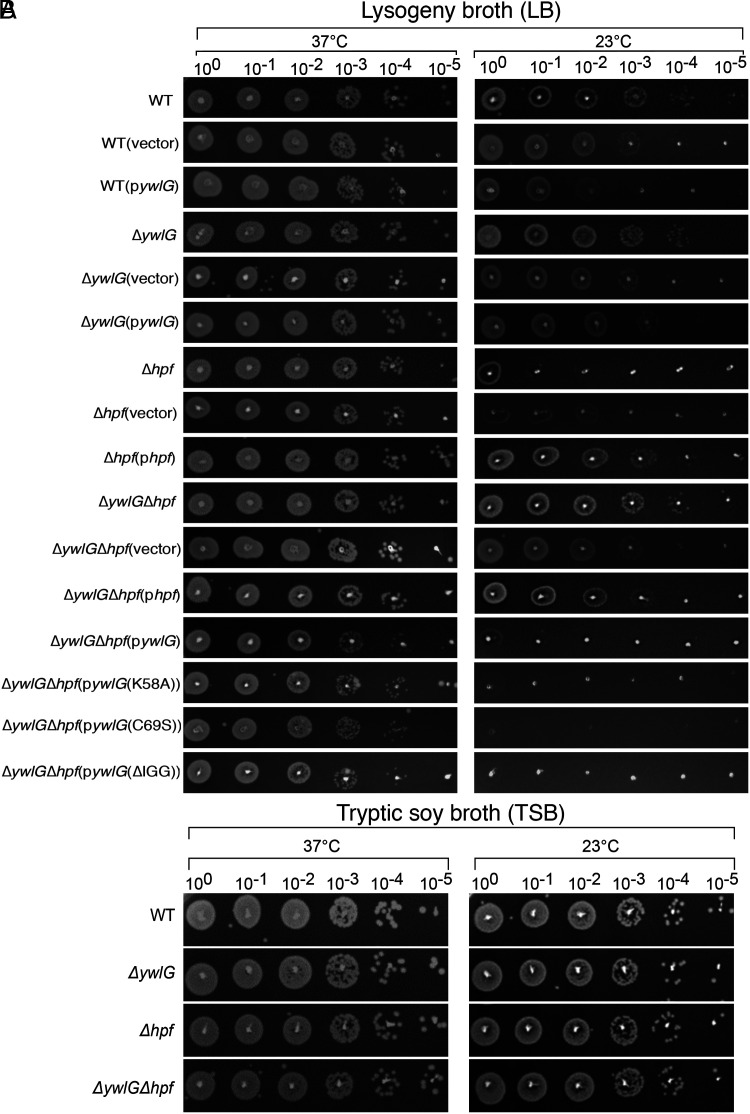
Inactivating *ywlG* suppresses the cold sensitivity of a Δ*hpf*-null mutant in a medium-dependent manner. Complementation with plasmid-encoded *ywlG* and *hpf* confirmed the YwlG-mediated suppression and HPF-dependent cold response. Serial dilution spot assays were performed on (*A*) lysogeny broth (LB)-based or (*B*) tryptic soy broth (TSB)-based agar plates. Exponentially growing cells (OD_600_ = 0.6) in TSB were adjusted to OD_600_ = 0.2 after two washes in 1xPBS. After serial dilutions, 2 µL of each dilution was spotted on the agar plates. The results were recorded after 24 h and 48 h of incubation at 37 °C and 23 °C, respectively. The images are representative of three independent experiments.

Cold sensitivity is a hallmark of defects in ribosome biogenesis and translation (36). It has been assumed that, at lower temperatures, rRNA folding is slowed, ribosome assembly is hindered, the enzymatic activity of translation factors is reduced, and mRNA secondary structure is increased. While a Δ*hpf*-null mutant does not exhibit measurable growth defects under various laboratory conditions, the growth of a Δ*hpf*-null mutant on LB agar at low temperature (23 °C) was reduced by four orders of magnitude, and the cold sensitivity could be fully rescued by expression of *hpf* in trans (Fig. 2*A*). These defects were not seen on a tryptic soy broth (TSB)-based agar (Fig. 2*B*). LB medium is known to undergo rapid depletion of carbon sources and to contain low concentrations of Mg^2+^ and Ca^2+^ (37, 38). The cold sensitivity of the Δ*hpf*-null mutant was likely associated with defects in ribosome assembly due to Mg^2+^ deficiency and/or loss of cell-membrane strength as a result of insufficient Mg^2+^ and Ca^2+^. Importantly, introducing Δ*ywlG*-null allele in the Δ*hpf*-null mutant fully restored the growth of Δ*hpf*Δ*ywlG* to the wild-type (WT) levels. Genetic complementation with an *hpf*-expressing plasmid rendered cold tolerance of the Δ*hpf*Δ*ywlG* double mutant, whereas introducing a *ywlG*-expressing plasmid to the Δ*hpf*Δ*ywlG* double mutant restored cold sensitivity (Fig. 2*A*). Furthermore, overexpression of YwlG in the WT background sensitized cells at low temperature by ∼10-fold, presumably due to insufficiency of HPF (partially resembles a Δ*hpf*) in excess of YwlG (Fig. 2*A*). Overexpression of YwlG in the WT also caused a growth delay (*SI Appendix*, Fig. S4*A*), implying that excess amount of YwlG is detrimental. Taken together, these results indicate that *hpf* mutation renders medium-specific cold sensitivity and that inactivation of *ywlG* suppresses the Δ*hpf* phenotype, providing evidence that *hpf* and *ywlG* are genetically and functionally linked, in addition to engaging in direct physical interaction.

### Inactivation of *ywlG* Promotes Stationary Phase Survival of Δ*hpf*-Null Mutant under Conditions of Excess Glucose.

**Fig. 3.**
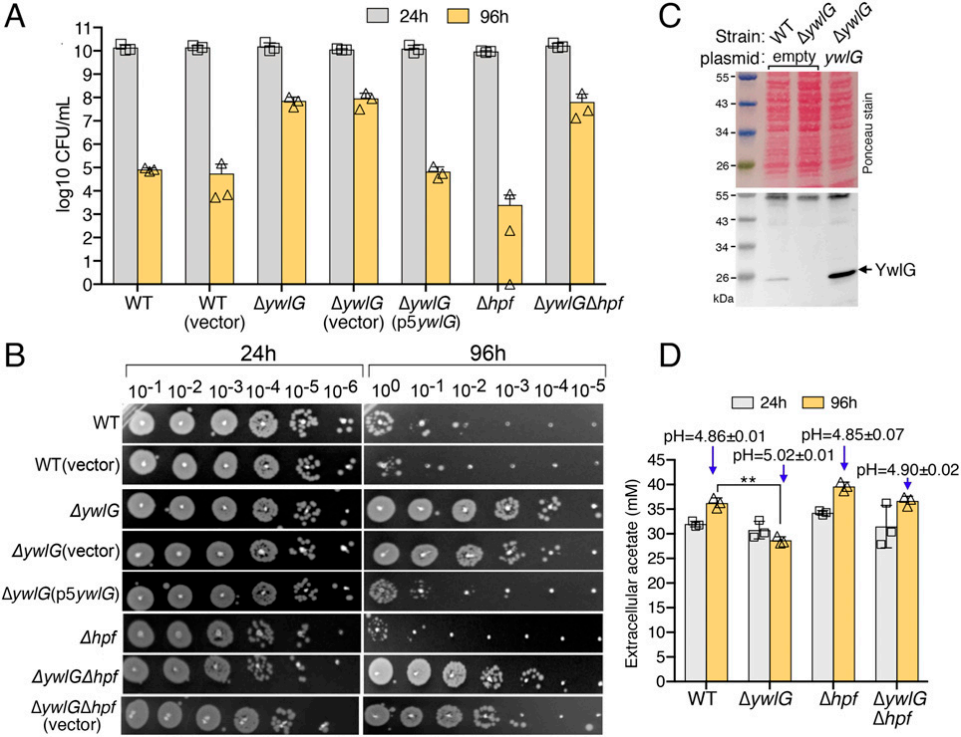
A Δ*ywlG*-null mutant is resistant to glucose-dependent cell death in the stationary phase. (*A*) Excess glucose differentially impacts cell death in *S. aureus* mutant strains. A Δ*ywlG* knockout promoted cell viability and suppressed cell death in a Δ*hpf*-null mutant. Cell viability (CFU/mL, *n* = 3, mean ± SD) was monitored over a period of 4 d in TSB-45 mM glucose. (*B*) Dilution spot assays for cells collected in *A*. Each spot corresponds to 2 µL of serially diluted cells. Genetic complementation was achieved by expressing *ywlG* from a plasmid. The empty vector served as a control. (*C*) Western blot showing the expression levels of endogenous YwlG (WT lane) versus plasmid encoded *ywlG* in TSB-glucose medium at 24 h. (*D*) Acetate levels and pH of culture supernatant of TSB-glucose cultures at 24 h and 96 h (*n* = 3, mean ± SD). A moderate reduction in acetate and a slight increase in pH were observed in the Δ*ywlG* backgrounds. **P < 0.01.

Aerobically grown *S. aureus* in TSB medium containing excess glucose (35–45 mM) enters stationary phase before the glucose is exhausted. However, bacteria continue to consume glucose and excrete acetate as a byproduct that, when not consumed as a secondary carbon source, potentiates cell death due to inflow of protonated acetate, which causes intracellular acidification and reactive oxygen species (ROS) production (39, 40). Motivated by these observations, we compared the survival of *Δhpf* and *ΔywlG* mutant strains in TSB-glucose medium by enumerating the colony forming units (CFUs) (CFUs/mL) (Fig. 3*A*) and by performing a dilution spot assay over a period of 4 d (Fig. 3*B*). Consistent with the findings of previous studies (39, 40), no significant differences in viable cell counts and growth rate were observed among all strains in the first 24 h of growth (Fig. 3 and *SI Appendix*, Fig. S4 *B* and *C*). However, the WT and a *Δhpf*-null mutant showed decreases of five and six orders of magnitude in cell viability after 96 h, respectively. In contrast, a *ΔywlG*-null mutant was more viable than the WT and the *Δhpf*-null strain by three and four orders of magnitude, respectively. Expressing *ywlG* from a plasmid converted the glucose tolerance of the Δ*ywlG*-null mutant into cell death. Furthermore, glucose killing in the *Δhpf*-null mutant could be suppressed by combination with a Δ*ywlG*-null mutant (Fig. 3 *A–C*). By an unknown mechanism, *Bacillus subtilis* YwlG is associated with 5-aminopentanoylation of the EF-P. EF-P functions to alleviate ribosome stalling at the polyproline sequence (41). That a Δ*efp* mutant did not phenocopy the Δ*ywlG* mutant (*SI Appendix*, Fig. S5) suggests that *S. aureus* YwlG and EF-P likely do not act in the same pathway during acetate-potentiated cell death. Finally, the degrees of cell death were only loosely correlated with acetate production and pH (Fig. 3*D*), suggesting that cytoplasmic acidification and pH were not the direct cause of cell death.

### The Δ*ywlG* Mutant Accumulates 100S Ribosomes.

**Fig. 4.**
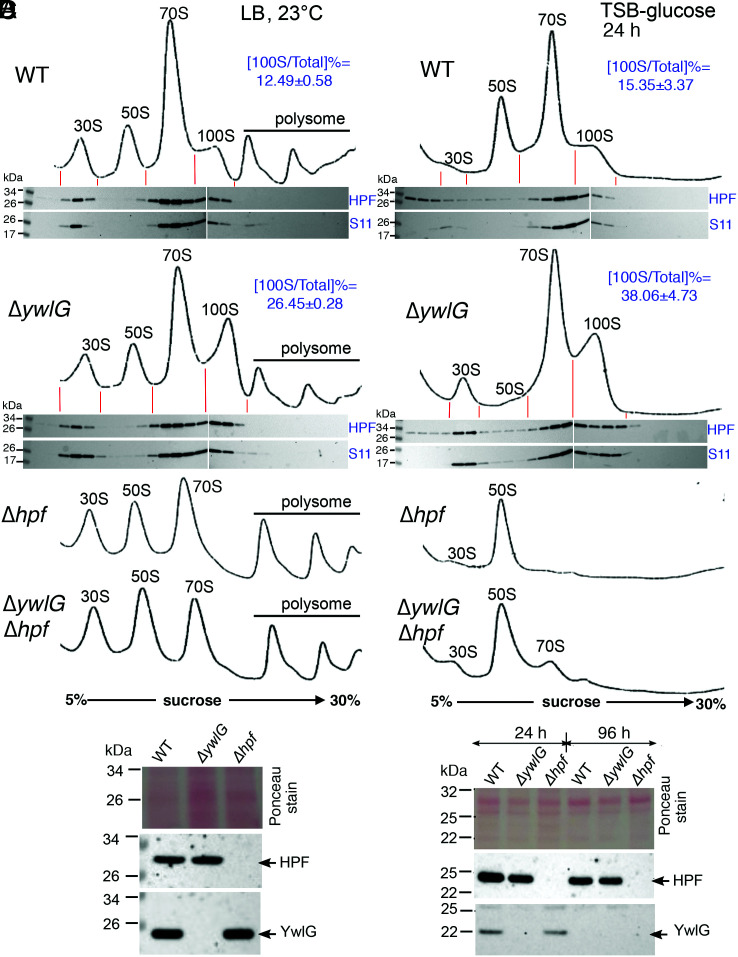
Inactivation of *ywlG* increases the formation of 100S ribosomes. (*A* and *C*) Ribosome sedimentation profiles of WT and its mutants. Cells were grown in LB at 23 °C until OD_600_ = 1.6 (*A*) or grown in TSB-glucose at 37 °C for 24 h (*C*). Crude ribosomes were extracted and subjected to 5–30% sucrose gradient fractionation (x axes), and ribosomal complexes were monitored by ultraviolet absorbance at 254 nm (y axes). Western blots show that significant amount of ribosome-free HPF was found in the WT than the Δ*ywlG* mutant (*C*, *Bottom*). The ratios of 100S ribosome versus total mature ribosome content were quantitated according to the areas under the curve using ImageJ (*n* = 3, mean ± SD). The 100S ribosomes accumulate upon *ywlG* deletion by greater than twofold relative to the level in the WT. (*B* and *D*) Western blots showing that the levels of HPF were unaffected by Δ*ywlG* knockout in the total lysates of LB cultures (*A*) and TSB-glucose cultures (*D*). Total proteins were resolved on a 4–12% Bis-Tris NuPAGE gel, and immunoblotting was performed with anti-YwlG (1/1,000) or anti-HPF (1/4,000).

To validate our initial hypothesis that YwlG sequesters HPF away from the ribosome binding, we examined the abundance of 100S ribosomes upon *ywlG* inactivation in LB (23 °C) and TSB-glucose cultures, two conditions that support the genetic and functional links between HPF and YwlG. Crude ribosomes were prepared by cryomilling to faithfully preserve ribosome complexes and translational states (13, 27). Next, we assessed the proportion of the 100S peak relative to the total mature ribosomes on a density gradient fractionation chromatogram. Under both conditions, the levels of 100S ribosomes in the Δ*ywlG* increased by twofold relative to the WT strain (Fig. 4 *A* and *C*). We confirmed that the accumulation of 100S ribosomes was not due to an increase in HPF concentration (Fig. 4 *B* and *D*). Since YwlG likely binds to the free HPFs not associated with ribosome (Fig. 1*F*), Western blots show that significantly more free HPFs were found in the low-density fractions in WT than in the Δ*ywlG* (Fig. 4*C*, *Bottom*). These results collectively indicate that HPF–YwlG binding prevents the formation of 100S ribosomes.

### The Δ*ywlG* Mutant Produces a Distinct Proteome under Excess Glucose Conditions.

**Fig. 5.**
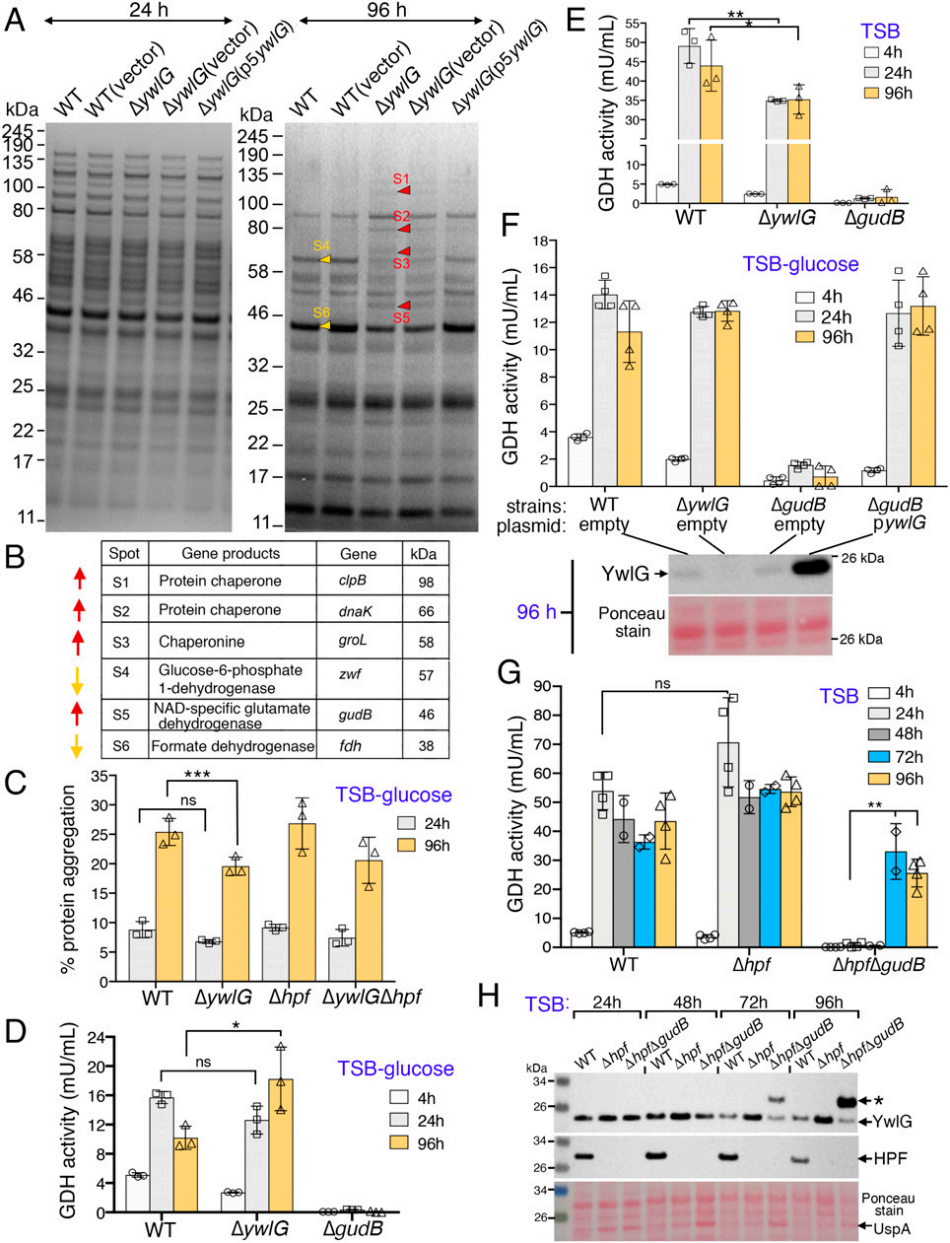
Proteomic approach to elucidate the biological activity of YwlG. (*A*) Comparative protein profiles of the WT, the Δ*ywlG* mutant, and the Δ*ywlG* complementation strain. Total proteins were extracted from TSB-glucose cultures at 24 and 96 h, resolved on a 4–12% Bis-Tris NuPAGE gel, and stained with EZ-Blue. Differentially expressed proteins are marked with red arrows (upregulated) or orange arrows (downregulated). The protein identities were determined by LC/MS-MS following in-gel trypsin digestion. (*B*) Differentially expressed proteins identified in *A*. (*C*) Strains containing the Δ*ywlG* mutant allele produce reduced amounts of protein aggregates. Total aggregates were measured with a PROTEOSTAT dye and the manufacturer’s supplied standards consisting of defined quantities of protein aggregates and nonaggregates. (*D* and *E*) Quantitation of glutamate dehydrogenase (GDH) activity in strains grown in TSB-glucose (*D*) and TSB (*E*). One unit of GDH is defined as the amount of enzyme that generates 1.0 µmol of NADH per minute at pH 7.6 at 37 °C. Excess glucose negatively impacted the production of GDH activity. (*F*) Overexpression of YwlG restored GDH activity in a Δ*gudB*-null mutant in TSB-glucose medium. Western blot showing the levels of YwlG protein at 96 h. (*G*) Combining the Δ*hpf* allele and a Δ*gudB* null mutant alleviated the inhibition of HPF on YwlG-mediated GDH activity in plain TSB medium after prolonged culture. (*H*) Western blot showing expression levels of YwlG and HPF in strains harvested from *G*. An asterisk indicates potential modification of YwlG or a nonspecific band against anti-YwlG. A universal stress protein homolog (UspA, SAUSA300_1656), as identified by MS, is upregulated in the Δ*hpf*Δ*gudB* mutant. ***P < 0.005, **P < 0.01, *P < 0.05, not significant (ns).

Beyond serving as an HPF sequestrator and participating in cold- and glucose-response pathways, the actual biological activity of YwlG remains unknown. We sought to address this knowledge gap by comparing the protein profiles of WT, Δ*ywlG*, Δ*hpf*, and their derivatives grown in TSB-glucose medium, a condition that produces one of the strongest phenotypes (Fig. 3). Total soluble proteins extracted from 24 h and 96 h were resolved on an SDS-PAGE gel, and differentially produced proteins were identified by mass spectrometry. Despite the low resolution and sensitivity of this one dimensional gel–based approach, we found that at least six protein species were differentially produced in the Δ*ywlG* and Δ*hpf*Δ*ywlG* strains at 96 h, whereas no differences were detectable at 24 h (Fig. 5*A* and *SI Appendix*, Fig. S6*A*), consistent with previous findings that all strains have similar viability at 24 h (Fig. 3*A*). The four upregulated proteins were the protein chaperones ClpB, DnaK, and GroL and the NAD-specific glutamate dehydrogenase GudB. Two metabolic enzymes, Zwf and Fdh, were downregulated (Fig. 5*B*). Importantly, the changes could be reversed by complementation with a plasmid encoding *ywlG*, confirming that these alterations were specific to the loss of *ywlG*. To rule out that the changes were technical artifacts, we validated the differential steady-state levels of one of the proteins (DnaK) by immunoblotting (*SI Appendix*, Fig. S6*B*).

The bacterial chaperone ClpB solubilizes and disaggregates misfolded proteins in conjunction with DnaKJE, and GroELS aids in protein refolding (42). The enrichment of ClpB, DnaK, and GroL in the Δ*ywlG* knockout mutant prompted us to test whether the loss of WT cell viability in TSB-glucose medium was due to accumulation of toxic protein aggregates. We used a thioflavin T–like analog, PROTEOSTAT, to quantitate the amount of protein aggregates. The analog is a molecular motor that spins in solution and produces no fluorescence. Intercalation of the analog into the amyloid fibril–like aggregates reduces rotation of the dye and generates strong fluorescence signals. Using this approach, we found that, indeed, strains bearing the Δ*ywlG* allele had ∼7% lower total protein aggregate amounts than the WT and Δ*hpf* strains (Fig. 5*C*). Although the effects were less pronounced than expected, we reasoned that an increase in the cell viability of the Δ*ywlG* knockout mutant in TSB-glucose medium could have occurred due to the derepression of protein chaperones, leading to alleviation of toxic aggregation and improvement of cell survival. To test this possibility, we introduced a Δ*clpB*-null allele into a Δ*ywlG* knockout mutant with the assumption that the double mutant would be less viable than the single Δ*ywlG* mutant. However, the Δ*clpB*Δ*ywlG* double mutant was equally viable compared with the single Δ*ywlG* mutant (*SI Appendix*, Fig. S5), suggesting that the accumulation of protein chaperones was likely a secondary byproduct or that inactivating one chaperone alone was insufficient to elicit cell death due to the redundancy of bacterial protein chaperones.

### YwlG Compensates for Loss of the NAD-Specific Glutamate Dehydrogenase GudB.

An ≃2.5-fold increase in GudB protein levels was observed in the Δ*ywlG* knockout mutant (Fig. 5*A*). GudB catalyzes the interconversion of glutamate to 2-oxoglutarate and ammonia while reducing NAD to NADH. Of note, the Δ*gudB* mutant was sensitive to glucose killing, similar to the WT and Δ*hpf* mutant (*SI Appendix*, Fig. S5). Using the Δ*gudB* mutant as a negative control, we performed glutamate dehydrogenase (GDH) activity assays to confirm that higher GudB in the Δ*ywlG* knockout mutant contributed to a modest increase in GDH activity in TSB-glucose medium at 96 h (Fig. 5*D*). In contrast, we found that GDH activity was moderately but significantly lower in the Δ*ywlG* knockout mutant than in the WT in plain TSB medium throughout the 96-h time course (Fig. 5*E*). Overall, GDH activity was >2.5-fold lower in all strains under conditions of excess glucose (compare y axes of Fig. 5 *D* and *E*), possibly due to CcpA-mediated catabolic repression of GDH genes similar to that reported in *B. subtilis* (43, 44) and *S. aureus* (45).

**Fig. 6.**
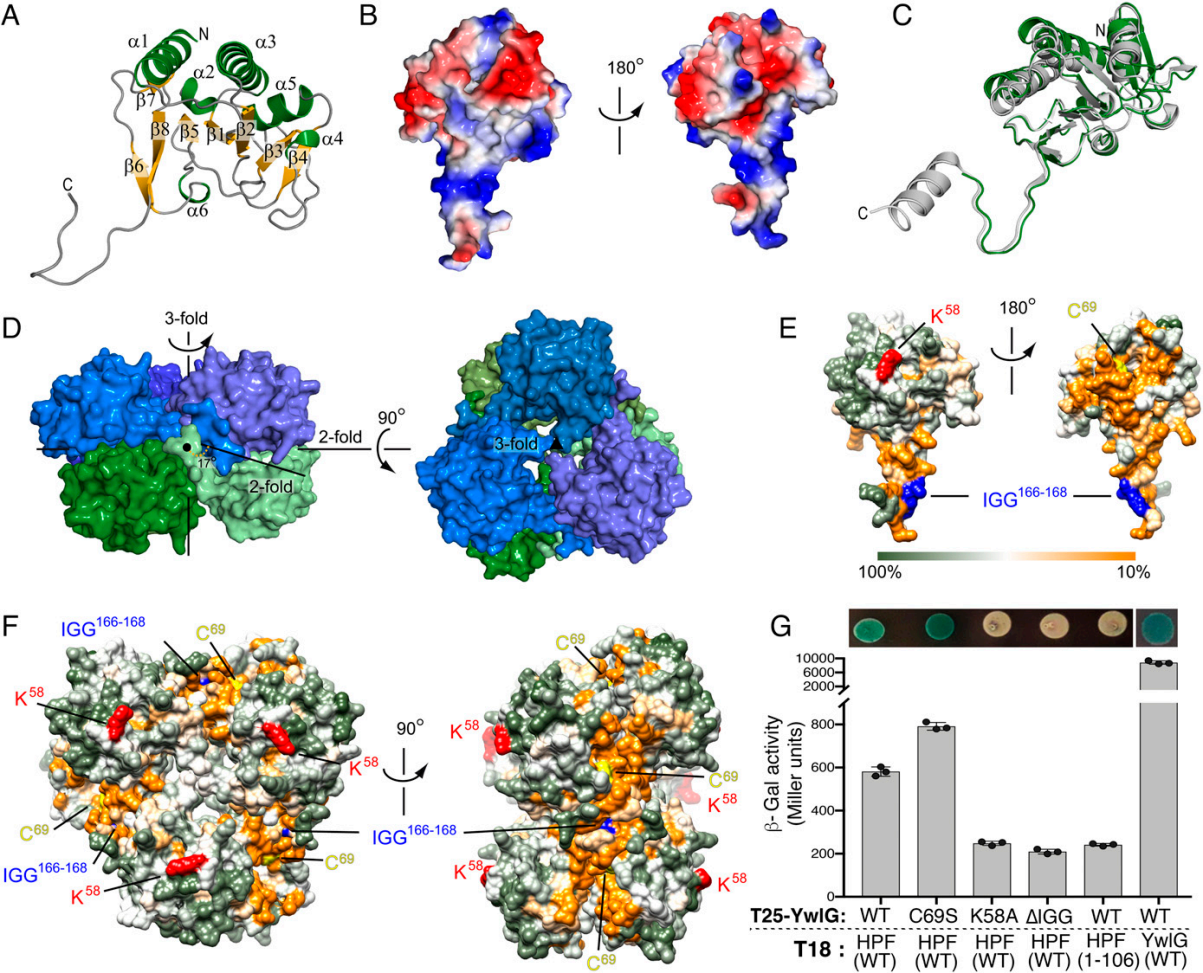
X-ray crystal structure of *S. aureus* YwlG and mutagenesis of YwlG and HPF. (*A*) Folding topology of YwlG shown on the monomer with secondary structural elements annotated. (*B*) Charges of amino acid side chains (red = negative; blue= positive) mapped onto the surface of a YwlG monomer. (*C*) Superposition of a YwlG monomer (green) with the homologous structure of the conserved hypothetical protein TT1679 from *Thermus thermophilus* (gray, PDB 1V8D). (*D*) Assembly of YwlG into a hexamer composed of two stacked trimeric rings. (*E* and *F*) Sequence conservation mapped onto the surface of the YwlG monomer (*E*) and hexamer (*F*), with K58, C69, and the ^166^IGG^168^ motif highlighted in red, yellow, and blue, respectively. (*G*) BACTH assay. HPF-CTD is important for HPF–YwlG interaction, whereas YwlG(K58A)-T25 and YwlG(ΔIGG)-T25 proteins are unstable (*SI Appendix*, Fig. S10), resulting in the lack of *β*-Gal activity. Interaction between YwlG-T25 and YwlG-18 independently validates the oligomerization status of YwlG in the crystals. The error bars indicate mean ± SD, *n* = 3.

Unlike *B. subtilis*, which carries two paralogous GDHs (RocG and GudB) (46), *S. aureus* possesses only GudB. Incidentally, *S. aureus* YwlG shares 11% sequence identity with the GudB homologs (*SI Appendix*, Fig. S7) and adopts a three-dimensional fold reminiscent of GDHs (Fig. 6). We hypothesized that YwlG and GudB may be functionally interchangeable, in part because we were unable to create a Δ*ywlG*Δ*gudB* double-knockout mutant despite numerous attempts. To test this hypothesis, we complemented a Δ*gudB* mutant with the plasmid-borne YwlG under control of *ywlG* native promoter. To our surprise, overexpressing YwlG in trans fully restored GDH activity to the WT levels in both TSB-glucose medium (Fig. 5*F*) and plain TSB (*SI Appendix*, Fig. S8*A*), while the mutant carrying the empty vector did not, implying that *S. aureus ywlG* may encode an alternative GDH.

If *S. aureus* YwlG processes GDH activity, the finding that the Δ*gudB* mutant completely lacked GDH activity suggests that YwlG activity might be repressed in the Δ*gudB* background, potentially due to the HPF–YwlG interaction. To test this possibility, we constructed a Δ*hpf*Δ*gudB* double mutant and repeated the GDH assays. In partial agreement with our prediction, a significant increase in GDH activity was detected in the Δ*hpf*Δ*gudB* mutant grown in prolonged TSB cultures (72–96 h) (Fig. 5*G*), but not in cells grown in TSB-glucose medium (*SI Appendix*, Fig. S8*B*). Immunoblotting showed an elevated YwlG protein level when *hpf* was deleted (Fig. 5*H*), suggesting that YwlG is destabilized by HPF in TSB. These results collectively suggest that YwlG is potentially a bona fide GDH that is only enzymatically active in the absence of HPF. Alternately, YwlG may serve as an activator of an otherwise silent GDH of unknown identity or is one of the components of an unidentified GDH complex (see Discussion).

### Structural Determination of *S. aureus* YwlG and Mutagenesis Analyses.

We determined the X-ray crystallography structure of YwlG to 1.75-Å resolution and did not detect bound PLP or other ligands. The data and refinement statistics are summarized in *SI Appendix*, Table S1. The fold of YwlG is characterized by a central eight-stranded antiparallel β-sheet decorated with three large and three short α-helices with an unusual 12-residue-long carboxyl-terminal extension (Fig. 6 *A–C*). The oligomeric state was determined by size-exclusion chromatography (SEC), in which YwlG was eluted with a size corresponding to a complex equivalent to a hexamer (*SI Appendix*, Fig. S9*A*). Notably, the elution volume is largely impacted by the shape of the protein or protein complex, with globular-shaped complexes traveling faster. Consistent with the SEC analysis, the asymmetric unit of the crystal was six protomers that made up two stacked trimers (Fig. 6*D*). The carboxyl-terminal extension appeared to interlock the oligomer. Analysis of the multimeric state of the protein showed that the hexamer was a stable composition, with a solvent-accessible surface area of 39,710 Å2 and a surface area upon hexamer formation of 12,540 Å2 (estimated G: −56.6 kcal/mol). The *S. aureus* YwlG monomer was almost superimposable on a conserved hypothetical protein from *Thermus thermophilus* (Protein Data Bank [PDB] 1V8D) (Fig. 6*C*), with which it shared 40% sequence identity (*SI Appendix*, Fig. S3). YwlG also shares distant similarity to GDHs, e.g., PDB 1V9L (47) and PDB 3K8Z (48). Notably, YwlG is half the size of the GDHs (*SI Appendix*, Fig. S7) and is only superimposable with the CTD of GudB1 (*SI Appendix*, Fig. S9*B*).

Guided by the YwlG structure, we attempted to delineate regions that are important for HPF interaction. We substituted the nonconserved residue K58 with an alanine because *S. aureus* YwlG K58 has been previously predicted to bind PLP (33). We replaced the invariant residue C69 with a serine because C69 was oxidized in the crystals with unknown significance. We deleted the ^166^IGG^168^ sequence in the carboxyl-terminal extension because the ^167^Gly-Gly^168^ dipeptide is invariant in all YwlG homologs (Fig. 6 *E* and *F* and *SI Appendix*, Fig. S3) and may be involved in trimeric oligomerization and/or HPF interaction. YwlG homologs are absent from bacteria harboring a short version of HPF that lacks the equivalent *S. aureus* HPF-CTD dimerizing domain (Fig. 1*A*). We predicted that HPF-CTD is responsible for interacting with YwlG because these proteins may have coevolved over time. We introduced a premature stop codon at the E107 position of HPF, which resulted in truncation of the entire CTD. These mutations were introduced into the existing BACTH system. With the exception of the YwlG(C69S) substitution, which resulted in an increase in β-galactosidase (β-Gal) activity, all other mutations produced negligible low β-Gal activity (Fig. 6*G*). Immunoblotting showed that the instability of CyaA’-YwlG(K58A) and CyaA’-YwlG(ΔIGG) in *E. coli* contributes to the lack of β-Gal activity, although the same YwlG mutants in the native *S. aureus* are fully functional in reversing cold sensitivity of a Δ*hpf* (Fig. 2*A*) and restoring GDH activity of a Δ*gudB* (*SI Appendix*, Fig. S8*C*). By contrast, the expression levels of CyaA’-HPF_1–106_ and CyaA’-YwlG(WT) were comparable (*SI Appendix*, Fig. S10); thus, the lack of β-Gal activity is due to the impaired HPF–YwlG interaction. YwlG–YwlG interaction was also validated in the BACTH system (Fig. 6*G* and *SI Appendix*, Fig. S10). While these experiments confirm the critical role of HPF-CTD in YwlG binding, more exhaustive separation-of-function mutagenesis will be needed to distinguish perturbation of protein oligomerization from defective partner binding.

## Discussion

**Fig. 7.**
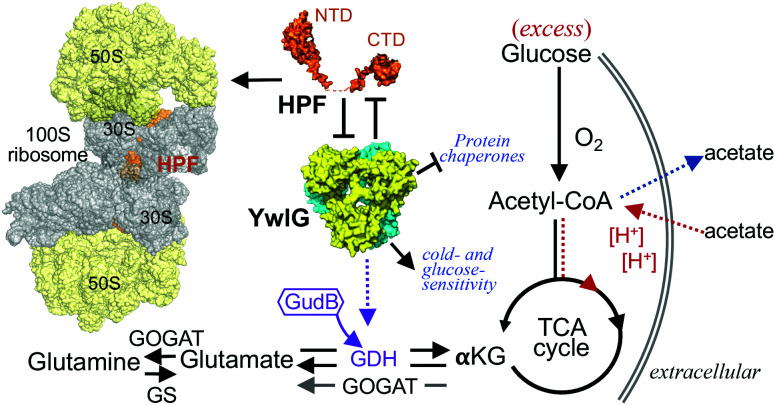
Interplay between HPF and YwlG. The carboxyl-terminal domain (CTD) of HPF promotes the dimerization of 70S monomers to form the translationally inactive 100S ribosomes. A fraction of HPF is sequestered by YwlG, diluting the levels of 100S complexes. Deletion of *ywlG* suppresses acetate-potentiated cell death and cold sensitivity in an *hpf* knockout, as well as derepresses the expression of protein chaperones. GDH resides at the crossroads of nitrogen- and carbon-assimilation pathways. YwlG structurally resembles distantly related NAD-specific GDHs and can functionally compensate for loss of the GudB GDH enzyme in *S. aureus* by an unresolved mechanism. A concentration threshold of YwlG is likely required for its GDH activity. GOGAT, glutamine oxoglutarate aminotransferase/glutamate synthase; GS, glutamate synthetase; *α*KG, *α*-ketoglutarate, Acetyl-CoA, acetyl-coenzyme A; TCA, Tricarboxylic acid cycle.

Bacterial hibernation factors are not known to interact with extraribosomal components. Here, we uncovered the genetic, physical, and functional links between the HPF and a previously uncharacterized YwlG protein in a MRSA. Two-way sequestration between HPF and YwlG is supported by (1) inactivation of *ywlG* suppressing cold sensitivity and glucose susceptibility of an Δ*hpf* knockout, (2) deletion of *hpf* derepressing YwlG-dependent GDH activity in a Δ*gudB* knockout, (3) HPF–YwlG interaction reducing the levels of 100S ribosomes, and (4) HPF without the carboxyl-terminal dimerization domain failing to interact with YwlG. Several major protein chaperones and metabolic enzymes were differentially expressed in the Δ*ywlG* mutant. Proteomic analysis, structural determination of YwlG, and functional complementation support a model in which YwlG is either a previously unrecognized GDH enzyme, a pseudo-GDH, or a stimulator of an unidentified GDH enzyme (*SI Appendix*, Fig. S8*D*). *S. aureus* YwlG has been found to be required for successful colonization in mouse models of pneumonia and osteomyelitis (34, 35). Our data reveal the moonlighting role of YwlG in regulating the abundance of 100S ribosomes (via HPF) and the important role of YwlG in glutamate metabolism (via GDH activity) (Fig. 7).

The precise orders of 70S ribosome dimerization and 100S complex disassembly are poorly understood. Consequently, how and when *S. aureus* YwlG outcompetes ribosomes for HPF remain to be determined. In *B. subtilis*, the resolution of 100S ribosomes is stimulated by active transcription that may involve competition between HPF and mRNA for ribosome binding (31). The identification of YwlG as the sequestrator of HPF provides an additional means to prevent the formation of translationally incompetent 70S dimers to meet the translational needs. Future studies focusing on the stoichiometry, stability, atomic structure, oligomeric state and kinetics of the HPF–YwlG interaction, as well as the timing of sequestration (pre-70S dimerization and/or post-100S dissociation) will offer a more holistic picture of the in vivo activity of the HPF–YwlG complex.

Very limited information is available about the biological activity of YwlG homologs aside from their possible roles in the modification of EF-P in *B. subtilis* (41) and PLP-dependent a.a. transformation (33). While these possibilities cannot be completely ruled out, a Δ*ywlG* and a Δ*efp* of *S. aureus* do not phenocopy each other, and PLP binding has not been validated in vivo or detected in vitro. The inability to detect PLP or other cofactor binding in the YwlG protein crystal could be due to instability of YwlG in its PLP-bound form or the missing component(s), e.g., HPF, in the heterologous *E. coli* from which recombinant YwlG proteins were isolated. Although *E. coli* harbors an HPF homolog, it lacks the hallmark self-dimerizing CTD uniquely found in YwlG-containing bacteria, implying a coevolution of YwlG and HPF-CTD.

The hexameric structure of *S. aureus* YwlG remotely resembles the structures of members of the catabolic, NAD-specific GDH family. Surprisingly, while GudB is the only known producer of GDH activity in *S. aureus*, overexpressing YwlG restored the GDH activity in a Δ*gudB*-null mutant, prompting our speculation that YwlG is a bona fide GDH. Further inspection of the protein domain structure revealed that YwlG did not conform to the conventional GDH domain assignment. First, YwlG is half of the typical size of a GDH and lacks the N-terminal catalytic domain and ligand binding sites (*SI Appendix*, Figs. S7 and S9*B*). Second, GDH activity was nearly undetectable upon *gudB* knockout, suggesting that YwlG alone was enzymatically inactive or that GDH activity was repressed by another factor in the Δ*gudB* mutant. One such repressor is HPF. However, inactivation of *hpf* led to only 50% derepression of YwlG-mediated GDH activity in prolonged TSB cultures, suggesting possible existence of an unknown repressor (*SI Appendix*, Fig. S8*D*). HPF and this repressor may sterically occlude GDH catalysis and substrate binding or prevent the formation of an active hexamer (46). Enzymatic repression of GDH via protein–protein interactions is not unprecedented. *B. subtilis* GudB is constitutively expressed, and its GDH activity is sequestered by the counter enzymes GltA–GltB under nutrient-specific conditions (49). A direct contribution of YwlG to GDH activity cannot be fully excluded. YwlG may serve as a pseudoenzyme to activate an unidentified GDH or may be a catalytically deficient GDH variant that requires a complementation fragment to fulfill GDH catalytic activity. The possible roles of YwlG in GDH activity are depicted in *SI Appendix*, Fig. S8*D*. Recombinant YwlG produced from *E. coli* by itself lacks dehydrogenase activity. Therefore, in vitro biochemical characterization of YwlG isolated from the native *S. aureus* is needed to discern all these possibilities.

The finding that the rescue of GDH activity in the Δ*gudB*-null mutant required YwlG overexpression suggests that the cellular concentrations of YwlG are critical in controlling GDH activity. Monomers and dimers of *B. subtilis* GudB are inactive. The oligomeric state of *B. subtilis* GudB is regulated by pH and glutamate levels (46). It is conceivable that overriding HPF repression and concentration-dependent hexameric assembly of YwlG drive GDH activity. *B. subtilis ywlG* is negatively regulated by the TnrA regulator in response to glutamine and ammonia availability (50). The regulation of *S. aureus ywlG* expression is unclear. An apparent TnrA binding motif is absent from the promoter region, and the YwlG protein, albeit not as abundant as HPF, was detectable under a wide range of laboratory conditions. YwlG appeared to be unstable in acidic environments in cells grown in TSB-glucose medium (Figs. 4*D* and 5*F*, barely detectable YwlG at 96 h), consistent with the notion that high pH promotes and/or stabilizes higher-order oligomers of GDH (46).

Cytoplasmic acidification causes *S. aureus* cell death under excess glucose conditions through generation of ROS (39, 40). Resistance to acetate-potentiated cell death in *S. aureus* carrying a Δ*ywlG* mutant allele may appear counterintuitive (Fig. 3). Glutamate is the major nitrogen reservoir and one of the most abundant cytoplasmic anions in bacteria, with cellular concentrations of up to 150 mM (51). Glutamate has multiple roles; for example, it stabilizes interactions among biomolecules (protein–protein interactions and protein–nucleic acid interactions), acts as a counterion to potassium, buffers intracellular pH, and maintains osmolarity (49, 51, 52). Glutamate can also be converted into the antioxidants glutathione (*S. aureus* produces bacillithiols instead) and 2-oxoglutarate. Our results suggest that YwlG contributes to glutamate metabolism via GDH activity. Although GudB is the core GDH in *S. aureus*, it is possible that moderate accumulation of 2-oxoglutarate upon Δ*ywlG* deletion provides some oxidative stress defense while not perturbing central GudB-dependent nitrogen and carbon metabolic circuits. An analogous anti-ROS survival strategy has been reported in a Δ*clpP* mutant that preserves antioxidant proteins otherwise degraded by the ClpXP protease (39).

Some eukaryotic and bacterial metabolic enzymes play dual roles as mRNA-binding proteins or ribosome-binding factors to modulate translation (53–55). In *B. subtilis*, the ribosome assembly checkpoint protein CpgA moonlights as a metabolite-proofreading enzyme (56). The discovery of bifunctional HPF and YwlG adds to a growing list of moonlighting proteins (57, 58) that regulate two of the most essential biochemical reactions (protein synthesis and glutamate metabolism) in all living cells.

## Materials and Methods

### Strains, Plasmids, Chemicals, and Growth Conditions.

Strains and plasmids were constructed as described in *SI Appendix* and are listed in *SI Appendix*, Table S2.

### FLAG Affinity Pull-Down and Mass Spectrometric Analysis.

*S. aureus* carrying the pLI50 derivatives were grown in 200 mL TSB supplemented with 10 µg/mL chloramphenicol at 37 °C until optical density at 600 nm equals 1.4 (4 h) or ≥8.0 (18 h). Cells were disrupted using cryomilling method (13) on a Retsch MM400 miller in Buffer C (50 mM Tris-Cl [pH 7.5], 100 mM KCl, 1 mM phenylmethylsulfonyl fluoride). Approximately 1 mL of cell lysates was incubated with 50 µL anti-FLAG M2 magnetic beads (Sigma-Aldrich, M8823-5ML) at room temperature (∼22 °C) on a tube rotator for 1 h and additional 15 h at 4 °C. The magnetic beads were washed extensively with Buffer C (7 ×1 mL), and proteins were eluted with 100 µL glycine (pH 2.6). Samples were neutralized with 15 µL 1 M Tris base and analyzed on a 4–12% Bis-Tris NuPAGE gel (Invitrogen). Liquid chromatography–tandem mass spectrometry (MS) was performed on trypsin digested gel slices or gel-free whole eluate by the Northwestern University Proteomics Core to identify protein species. Scaffold (version Scaffold_4.11.0, Proteome Software Inc., Portland, OR) was used to validate MS/MS-based peptide and protein identifications.

#### Common molecular biology techniques.

BACTH system, ribosome sedimentation profiling, Western blots, TSB-glucose killing assays, quantitation of acetate and protein aggregates, and recombinant YwlG production are described in *SI Appendix*.

### GDH Activity Assay.

One milliliter of bacterial cultures was collected, resuspended in sterile water, and lysed on a FastPrep-24 instrument using Lysing Matrix B beads (MP Biomedicals), and 10 µL cell lysates were immediately used in the reactions. GDH activity was measured by the amount of NADH production upon glutamate consumption using a colorimetric kit according to manufacturer’s procedures (Sigma-Aldrich No. MAK099). One unit of GDH is defined as the amount of enzyme that generates 1.0 µmol of NADH per minute at pH 7.6 at 37 °C.

### YwlG Crystallization and Structural Determination.

Overexpression and purification of recombinant YwlG are described in *SI Appendix*. Prior to crystallization, YwlG was centrifuged (16,000 × *g*, 4 °C, 20 min) to remove aggregates and debris. Diffraction-quality crystals of *S. aureus* YwlG were obtained using the sitting-drop vapor diffusion in 0.2 M Na^+^K^+^ tartrate, 20% (wt/vol) PEG3350 and 0.18 M ammonium citrate, 20% (wt/vol) PEG3350 at 18 °C. For cryoprotection, crystals were soaked in mother liquor supplemented with 30% (wt/vol) ethylene glycol, flash cooled, and stored in liquid nitrogen. Data collection was carried out at the synchrotron beamline ID30B and ID30A1 at the European Synchrotron Radiation Facility, Grenoble, France. The data were processed with XDS (59), and the structure was solved by molecular replacement with PHASER (60), using the coordinates of the conserved hypothetical protein TT1679 from *T. thermophilus* (PDB 1V8D), which shares about 36% and 52% sequence identity and similarity, respectively, with YwlG from *S. aureus*, as a search model. Model building was done with COOT (61), and refinement of the coordinates was carried out with REFMAC5 (62, 63). The hexameric complex was analyzed using PdbePISA (64). Structural figures were prepared with PyMOL (65) and University of California, San Francisco Chimera (66).

### Statistically Analysis.

Statistical significance was tested by unpaired two-tailed Student’s *t* test in GraphPad Prism 9.0. Only *P* < 0.05 is considered statistically significant.

## Supplementary Material

Supplementary File

## Data Availability

Crystallographic data (atomic coordinates and structure factors) of YwlG have been deposited in the PDB with accession code 7Z06 (https://www.ebi.ac.uk/pdbe/entry/pdb/7Z06) (67). [Protein structure] data have been deposited in [RCSB PDB] (7Z06; https://www.ebi.ac.uk/pdbe/entry/pdb/7Z06). All other data are included in the manuscript and/or *SI Appendix*.
