# Supplementary material for: Bidirectional sequestration between a bacterial hibernation factor and a glutamate metabolizing protein
Source: Proc Natl Acad Sci U S A. 2022 Sep 19;119(39):e2207257119. doi: 10.1073/pnas.2207257119 (PMC9522360; doi:10.1073/pnas.2207257119)
Supplement: Supplementary File [file pnas.2207257119.sapp.pdf]

PNAS

Supplementary Information for

Bidirectional sequestration between a bacterial hibernation factor and a glutamate metabolizing protein

David Ranava[1], Christopher M. Scheidler[2], Martin Pfanzelt[3], Michaela Fiedler[3], Stephan A. Sieber[3#], Sabine Schneider[2#], and Mee-Ngan F. Yap[1#]

[1] Department of Microbiology-Immunology, Northwestern University Feinberg School of Medicine, Chicago, Illinois, USA.

[2] Department of Chemistry, Ludwig-Maximilians-Universität München, Butenandtstr. 5-13, 81377 Munich, Germany.

[3] Department of Chemistry, Chair of Organic Chemistry III, Center for Functional Protein Assemblies (CPA), Technische Universität München, Garching, Germany.

[#] Corresponding authors

Email: frances.yap@northwestern.edu (Lead Contact)
Email: sabine.schneider@cup.lmu.de
Email: stephan.sieber@tum.de

This PDF file includes:

SI Materials and Methods
Figures S1 to S10
Tables S1 to S3
SI References

SI Materials and Methods

Strains, plasmids, chemicals, and growth conditions.

Strain JE2 is a community-associated methicillin-resistant *Staphylococcus aureus* (CA-MRSA) of USA300 lineage (1). The JE2 mutant derivatives carry a *bursa aurealis* transposon insertion were acquired from BEI Resources and were backcrossed to isogenic JE2 background by **Φ**11 phage transduction. When necessary, erythromycin (Erm) resistance cassette of the transposon mutants was swapped with a spectinomycin (Spc) resistance marker according to the established protocols(1). The desired Spc^R mutant allele was introduced to the destination JE2 derivatives by **Φ**11 transduction and confirmed by inverse PCR coupled Sanger sequencing. All strains and plasmids are listed in Table S2.

The in-frame *efp* deletion mutant (MNY249) was constructed as follows: A 2-kb flanking region of the *efp* (locus SAUSA300_1490) was PCR amplified with the primer pairs P1591/P1592 and P1593/P1594 via 2-step PCR using JE2 genomic DNA as the template. The product was digested with SacI and SalI and cloned into the same sites of pBT2 (2). The resulting pBT2Δ*efp* was digested with SmaI, dephosphorylated, and ligated to the blunt-ended a 1.6-kb kanamycin (Km) resistance cassette that was released from pBTK (3) by KpnI and PstI digestion. The resulting construct pBT2Δ*efp*::Km was propagated in *E. coli* DC10B, and the plasmid reisolated, electroporated into JE2 and selected at 30°C on agar plates supplemented with 10 µg/mL chloramphenicol. The integrant was furthered selected by a 43°C temperature upshift on chloramphenicol-containing agar plates. The homologous recombinant was resolved by 30°C passages and cycloserine enrichment following the published procedures (3).

To construct p*ywlG* bearing *ywlG* native promoter, primers P1659 and P1660 were used to amplify the region using JE2 genomic DNA as a template. The product was cloned into the KpnI and HindIII sites of pLI50. Restriction-free cloning (4) was used to introduce 2×FLAG into p*hpf* (5) to generate $pHPF^{2\times FLAG}$ and to assemble *ywlG* onto a xylose-inducible pEPSA5 (named p5y*wlG*) (6). Site-directed mutagenesis was performed using Quikchange mutagenesis kit (Agilent Genomics). BACTH plasmids (Euromedex) carrying the YwlG-T25, YwlG-Y18 and T18-HPF fusion were constructed by the restriction-free cloning (4). Primers were purchased from IDT DNA and are listed in Table S3. Sanger DNA sequencing was performed by Genewiz-Azenta Life Sciences.

Unless otherwise noted, *S. aureus* cells were grown aerobically at 37ºC in tryptic soy broth (TSB, BD Difco™#211822), lysogeny broth (LB, BD Difco™ #244610), or dextrose-free TSB (BD Difco™ #286220) supplemented with 45 mM glucose (TSB-glucose) at a 5:1-10:1 tube- or flask-to-medium ratio with a 1:100 dilution of an overnight seed culture. *E. coli* were grown in LB. When necessary, erythromycin, chloramphenicol, kanamycin, ampicillin, xylose, spectinomycin and IPTG were used at 5 µg/mL, 5-10 µg/mL, 75-100 µg/mL, 100 µg/mL, 10 mM, 1 mg/mL and 0.5 mM, respectively. All chemicals were from Sigma-Aldrich unless otherwise noted.

Bacterial 2-hybrid system (BACTH).

pKT25 and pUT18C derivatives were co-transformed into *E. coli* BTH101. β-galactosidase activity was determined as described previously (7). The specific activity was expressed in Miller Units (8). In parallel, LacZ production was visualized on LB agar supplemented with 40 µg/mL X-Gal (5-Bromo-4-chloro-3-indolyl beta-D-galactopyranoside, ThermoFisher) and 0.5 mM IPTG after 24-30 hr incubation at 30°C.

Ribosome sedimentation profiles.

Crude ribosomes were isolated from *S. aureus* by cryo-milling methods (5) in Buffer A [20 mM HEPES (pH 7.5), 14 mM $Mg(OAc)_2$, 100 mM KCl, 0.5 mM PMSF, 1 mM DTT] (5, 9). Five absorbance units (Abs_{260}) of ribosomes were layered on a 5-30% sucrose gradient that was prepared on a BioComp Gradient Master. The samples were centrifuged at 210,000 ×*g* at 4°C in a SW41 rotor in a Beckman Coulter Optima XPN-100 ultracentrifuge for 3 hr. Fractionation was performed using a Brandel fractionation system equipped with a UA-6 UV detector. To quantitate the abundance of 100S ribosomes relative to the total mature ribosomes, the boundaries of ribosomal peaks were manually selected from the trough between the peaks. The total area under a peak was calculated by ImageJ and divided to obtain the ratio. When immunoblotting was needed, ~200 µL per fraction were collected and subjected to final 10% trichloroacetic acid

precipitation. The pellets were washed with cold acetone once, resuspended in 50 mM Tris base containing Laemmli sample buffer and resolved on a 4-20% TGX SDS-PAGE (BioRad).

TSB-glucose killing assays.

Strains were grown in dextrose-free TSB supplemented with 45 mM glucose at 37°C and 250 rpm with aeration in a flask-to-medium ratio of 10:1 for 4 h-96 h. Serial dilutions were carried out with sterile 1×PBS and plate on TSB-agar. Colonies were enumerated after 24 h incubation at 37°C. For spot assays, 2 µl of each diluted cell suspension was spotted on TSB-agar and incubated overnight at 37°C.

Antibodies and Western Blots.

S. aureus cell pellets were homogenized with Lysing Matrix B (MP Biomedicals) in 25 mM Tris (pH 7.5) on a FastPrep-24 homogenizer (MP Biomedicals). Clarified lysates were recovered by spinning at 20,817×*g* at room temperature for 5 min to remove cell debris. A total of 0.1-0.2 Abs_{280} units of cell lysate were analyzed on 4-20% TGX SDS-PAGE gels (BioRad), or 4-12% Bis-Tris NuPAGE minigels (Invitrogen) and the proteins were transferred to a nitrocellulose membrane using a Trans-Blot Turbo system (BioRad). The membrane was stained with Ponceau red (Amresco #K793-500mL) to ensure equal loading, followed by immunoblotting using anti-YwlG (1:1,000 dilutions), anti-S11 (1/4,000 dilutions), anti-HPF (1/4,000-1/8,000 dilutions), anti-FLAG M2 (1/1,000, Cell Signaling #2368), anti-DnaK MAb(8E2/2) (Enzo #ADI-SPA-880-F, 1/1,000 dilutions), anti-CyaA (Santa Cruz #sc-13582, 1/1,000 dilutions). Polyclonal rabbit anti-S11 (10) and anti-HPF (9) were generated and described previously. To generate anti-YwlG, two peptides corresponding to (9-23) and (157-174) residues of the *S. aureus* YwlG [Cys-^{9}DELKDMSFFNKGDIC^{23}; Cys-^{157}TIATSRPKKIGGERAKYQ^{174}] were custom synthesized and used for immunization in New Zealand white rabbits (Pacific Immunology®). HRP-conjugated anti-IgG secondary antibody (1/15,000 dilutions) was from Cytiva (# NA9120, 1/15,000 dilutions). HRP-linked anti-mouse secondary antibody was from ThermoFisher Scientific (#62-6520,1/10,000 dilutions). SuperSignal™ West Dura chemiluminescence substrate was used (Thermo Scientific #34075). Images were acquired using iBright FL1500 system (ThermoFisher).

Quantification of acetate.

Extracellular acetate concentrations of TSB-glucose cultures were measured using commercially available colorimetric kits (Sigma-Aldrich MAK086) according to the manufacturer's protocols. One milliliter of cells was harvested at given time points by centrifugation at 18,407×g for 5 min. The supernatant was passed through a 0.2 µm syringe filter to remove residual cells. Ten microliters of supernatant were used for the quantification of acetate using a standard provided in the kit.

Quantification of protein aggregates.

Total cellular protein aggregates were determined by a fluorescence based PROTEOSTAT® assay kit (Enzo #ENZ-51023-KP002) using the manufacturer's provided standards. PROTEOSTAT® is protein aggregates sensitive fluorescent dye. The standards contain a defined quantity of protein aggregates and soluble proteins. One milliliter of TSB-glucose cultures was collected at indicated time point (18,407×*g*, 10 min), cell pellets were washed once with 1×PBS, resuspended in 1×PBS, and disrupted on a Fastprep-24 homogenizer (MP Biomedicals) for 2 cycles of 40 sec at speed of 4M/sec using Lysing Matrix B beads (MP Biomedicals). The relative amount of protein aggregates was determined by the standard curve according to manufacturer's instructions. Ten microliters of samples were used in each reaction. Fluorescence intensity was measured on a Spark® multimode microplate reader (Tecan) with a filter set of an excitation at 550 nm and an emission at 600 nm. Readings were taken in at least duplicate in a Greiner® µClear black, clear bottom 96-well microplate.

Purification of recombinant YwlG

E. coli BL21(DE3) cells were transformed with the overexpression plasmid (pDEST-Q2FF14) encoding the N-terminally StrepII-tagged YwlG protein. Cells were grown overnight in LB medium supplement with 50 µg/ml kanamycin at 37°C and 180 rpm. Cells from the overnight cultures were diluted to a final OD_{600} of 0.05 in 2YT medium (50 µg/ml kanamycin) and incubated at 37°C and 180 rpm until an OD_{600} of 0.6 was reached. After

incubation on ice for 20 min, the protein expression was induced with 0.75 mM IPTG and the cells further grown overnight at 25°C, 180 rpm. Cells were subsequently harvested by centrifugation (5,000×*g*, 20 min; 4°C). Cells corresponding to 1L of expression culture were resuspended in 20 ml lysis buffer (50 mM Tris-HCl, 250 mM NaCl, pH 8.0) supplemented with 1 mg/mL DNase I (AppliChem), 0.1 mg/mL lysozyme (~20,000 U/mg; CarlRoth) and one tablet cOmplete^{TM} ULTRA EDTA-free protease inhibitor tablet (Roche). Cells were lysed by homogenization using an EmulsiFlex-C5 (Avestin, Inc.) and cleared lysate was obtained after centrifugation (24,446×*g*; 30 min; 4 °C) and filtration using a Whatman^{TM} folded filter (Cytiva). YwlG was purified in two-steps using an ÄKTA Pure system (Cytiva): First affinity purification taking advantage of the N-terminal StrepII-tag was using a 1 ml StrepTrap column (Cytiva) was carried out. After sample application the column was washed with 10 CV lysis buffer. YwlG was eluted using 10 CV elution buffer (50 mM Tris-HCl, 250 mM NaCl, 2.5 mM Desthiobiotin, pH 8.0). Fractions containing the protein were pooled and concentrated (Amicon Ultracell Centrifugal filter unit; MWCO 10 kDa, Merck Millipore) and applied to a Superdex 200 increase 10/300 GL column (Cytiva), equilibrated with 50 mM Tris-HCl, 250 mM NaCl, pH 8.0. Fractions containing YwlG were pooled, concentrated to 10-11 mg/mL and directly used for crystallization trials or flash-frozen in liquid nitrogen.

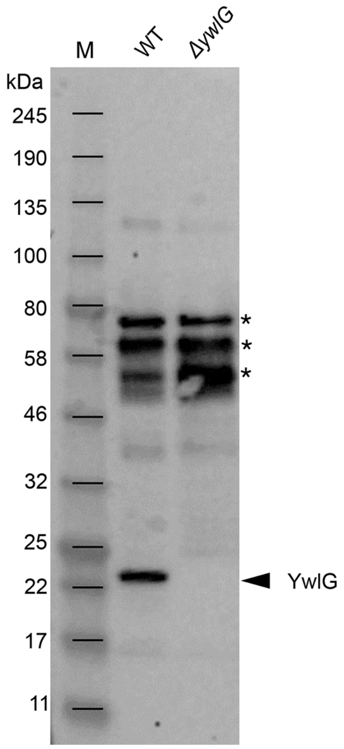

Figure S1. Western blot showing the specificity of the polyclonal anti-YwlG antibody. Immunoblot of YwlG in the *S. aureus* JE2 wild type (WT) and its Δ*ywlG*::*erm* null mutant. The asterisks indicate nonspecific cross-reactions. Purified anti-YwlG was used at a 1:1000 dilution. M marks the prestained protein standard (New England Biolabs #P7712).

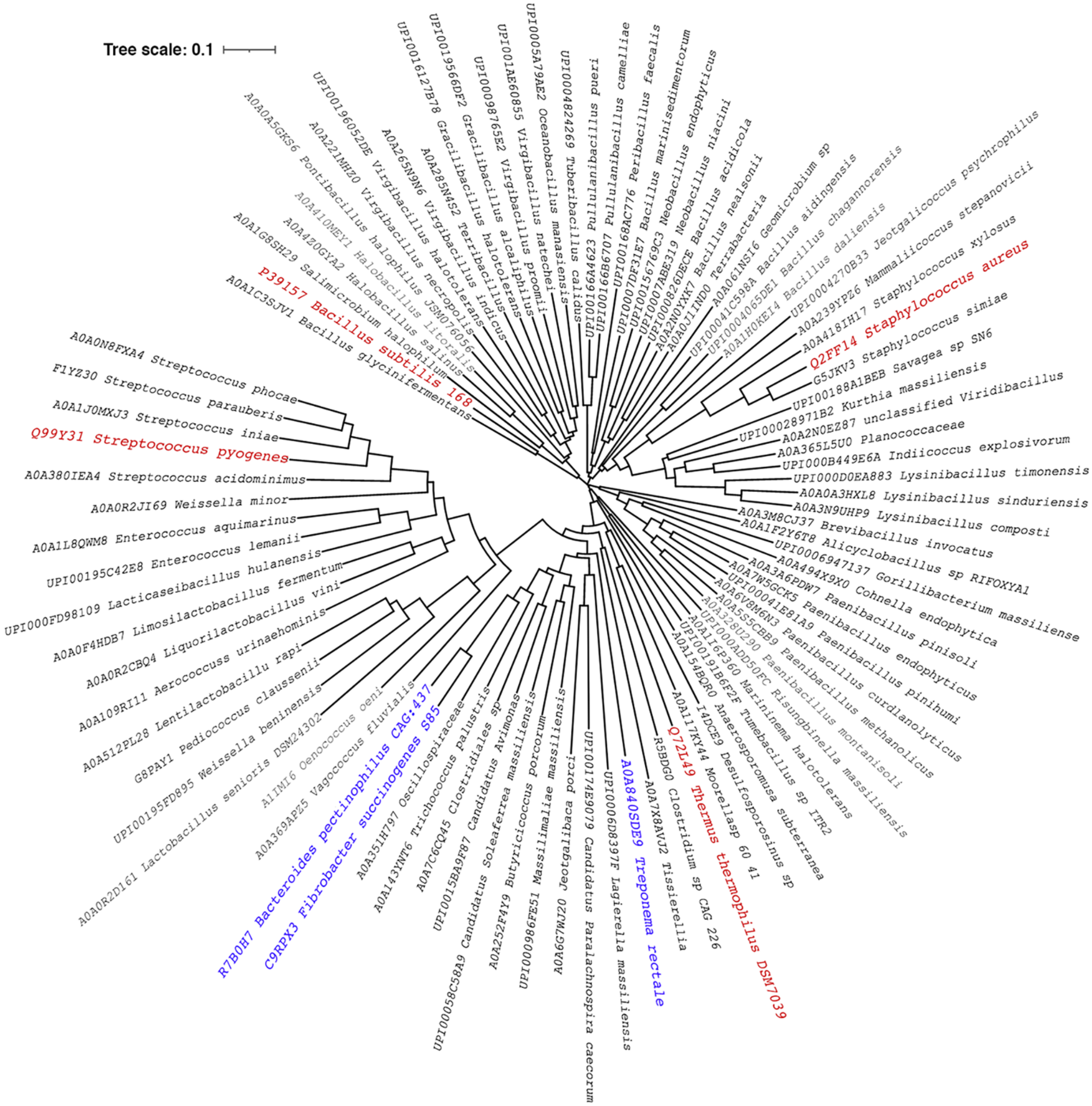

Figure S2. Circular dendrogram showing the prevalence of YwlG homologs in Firmicutes. The tree is based on 150 protein sequences (out of 256 homologs) of representative phyla. Sequence alignment was performed by ConSurf (11) using Bayesian analyses. A partial phylogenetic tree was built using the iTOL tool (12). Species representing Fibrobacteres, Bacteroidetes and Spirochaetota are highlighted in blue. Representative Firmicutes and Thermus spp. are colored in red. All the remaining species (in black) are Firmicutes of animal and human origins or free-living bacteria. The numbers indicate either the UniProtKB or GenBank accession numbers.

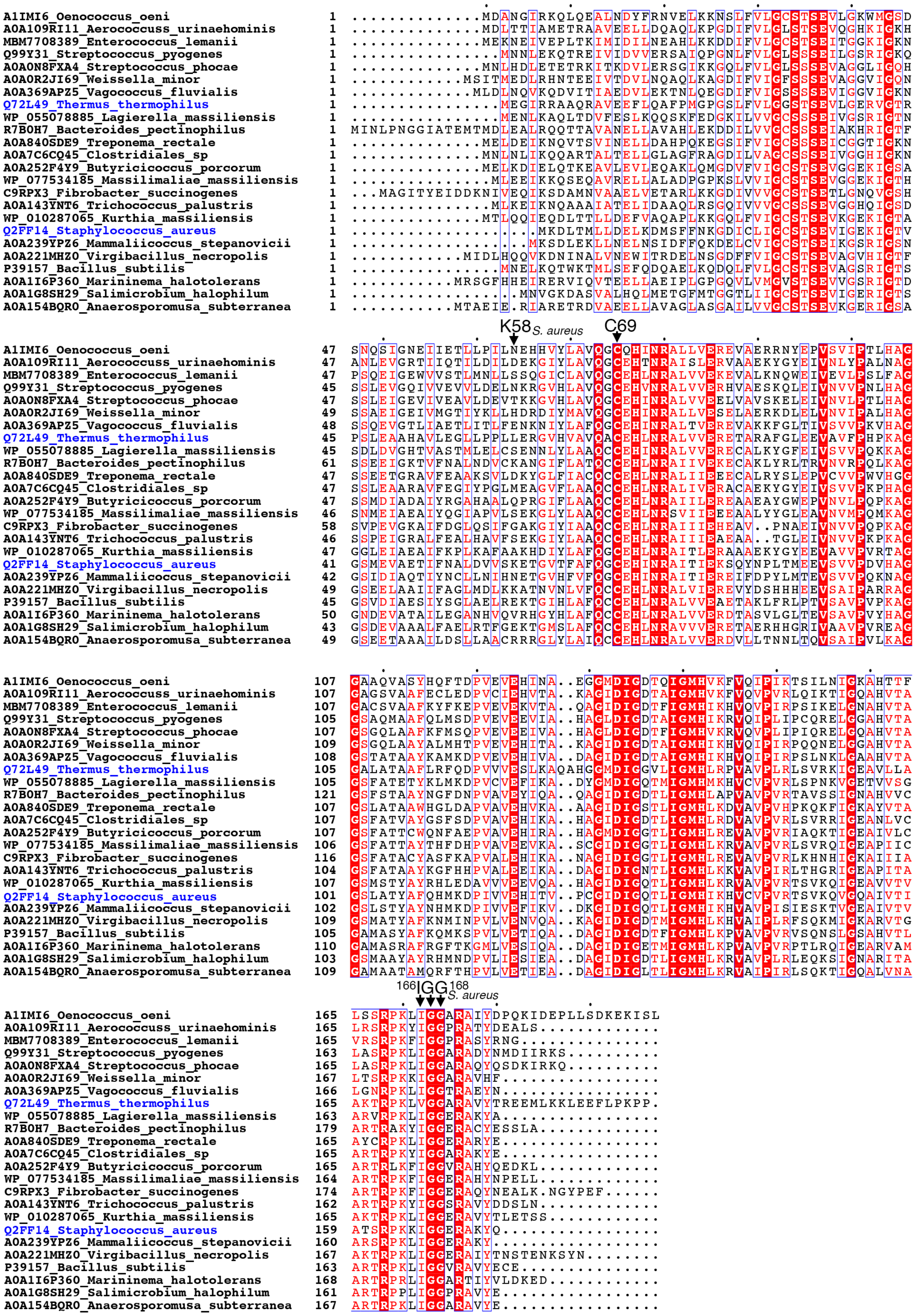

Figure S3. Multiple sequence alignment of YwlG homologs from representative phyla. Protein sequences were extracted from NCBI GenBank or UniProtKB with the indicated accession numbers. The sequences were aligned with Clustal Omega (13) and processed using ESPript 3.0 (14). The putative pyridoxal phosphate (PLP) binding site (K58), oxidized C69 and conserved IGG motif of YwlG are marked with arrows. Bacteria with solved atomic YwlG structures are colored blue.

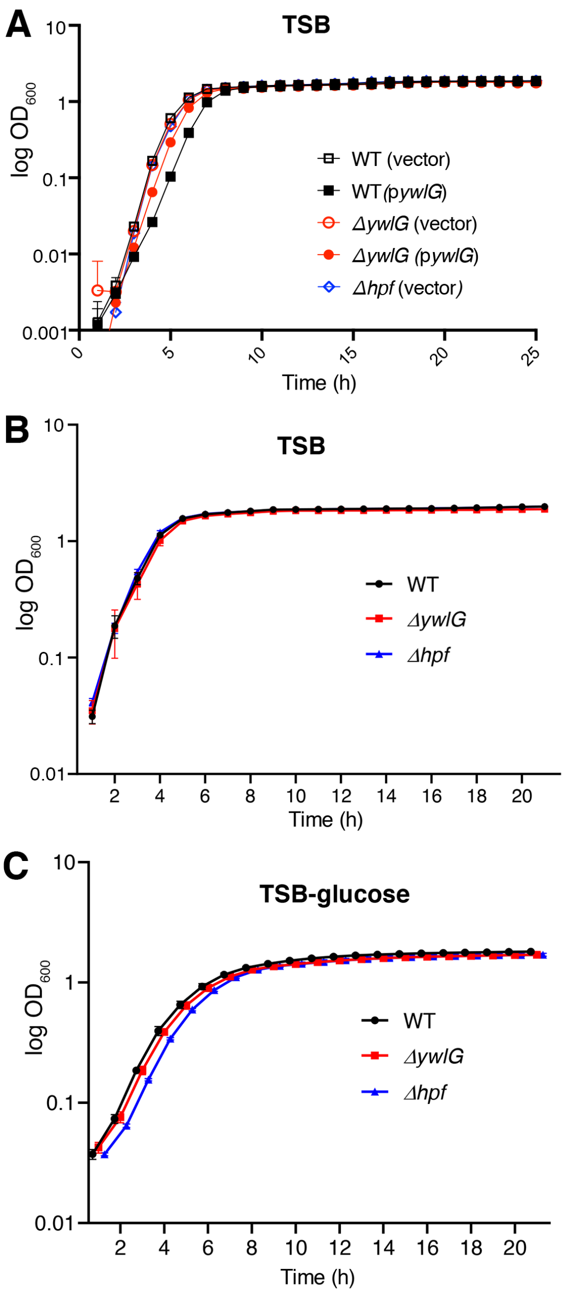

Figure S4. Growth kinetics of wild-type (WT), Δ*ywlG*, and Δ*hpf* strains in TSB or TSB-glucose media. (A) Overexpression of YwlG on a multicopy plasmid slows growth of WT cells. Chloramphenicol was added to a final concentration of 5 µg/mL to maintain the plasmids. **(B-C)** A Δ*ywlG* null mutant does not exhibit measurable growth defects in tryptic soy broth (TSB) and TSB supplemented with 45 mM glucose (TSB-glucose). The optical density (OD_{600nm}) was measured on a Tecan SPARK® microplate reader equipped with a humidity chamber at 37°C. The error bars indicate mean± SD, n=3.

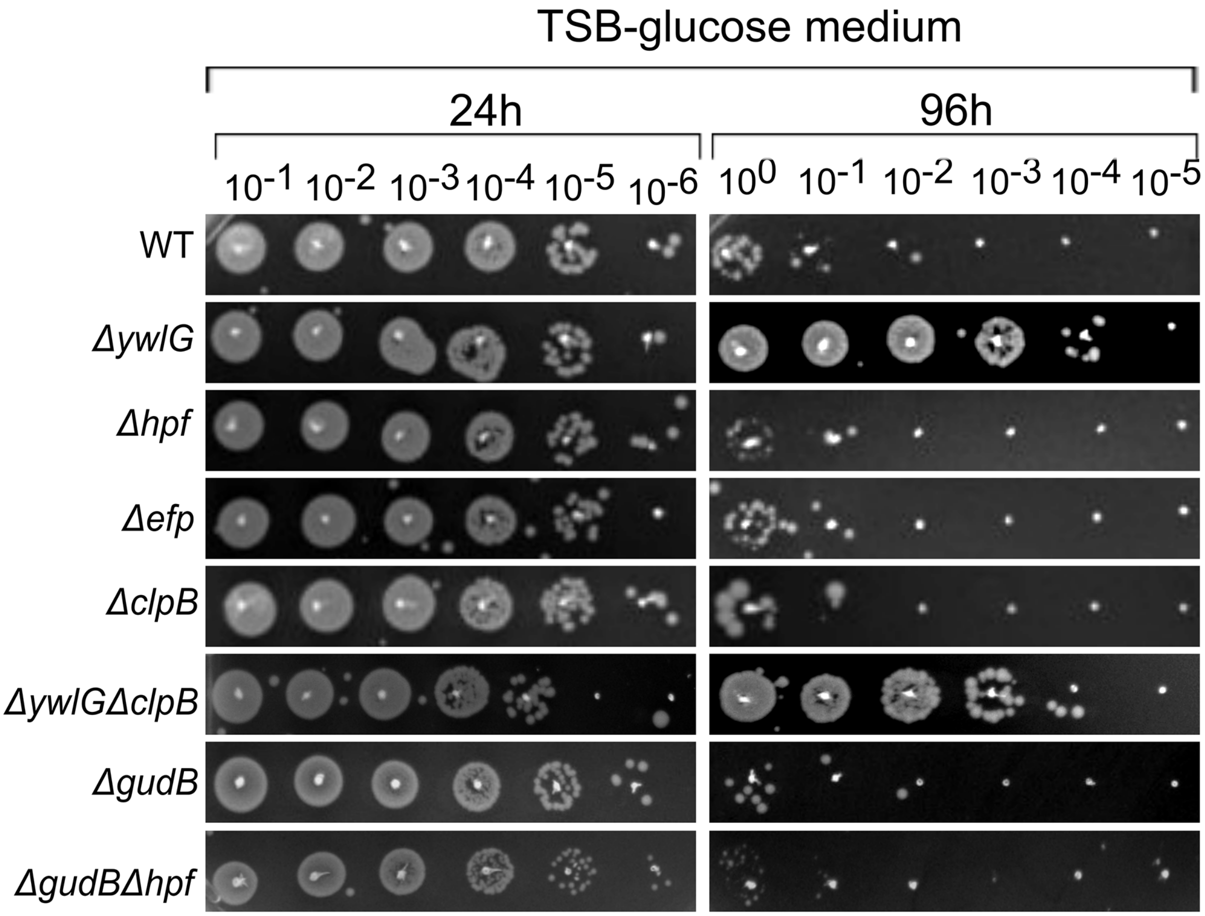

Figure S5. Dilution spot assays showing the differential cell viability of various *S. aureus* strains grown in TSB supplemented with excess glucose (45 mM). Inactivation of *ywlG* renders glucose resistance, whereas ∆*hpf*, ∆*efp*, ∆*clpB*, and ∆*gudB* null mutants are as sensitive as the wild type (WT) after 96 h at 37°C.

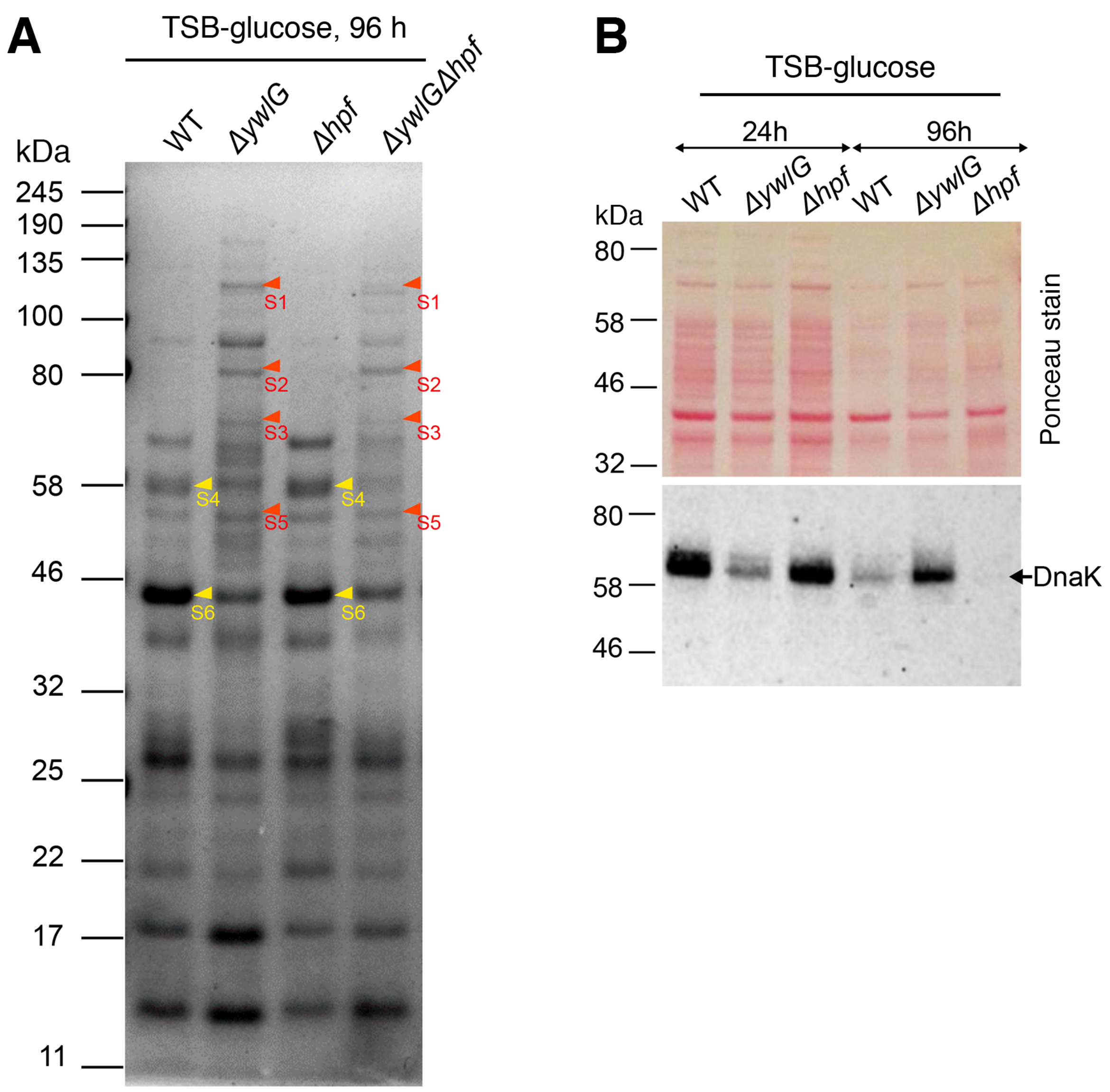

Figure S6. Comparative protein profiles of the wild-type (WT) strain and the Δ*hpf* and Δ*ywlG* mutants. (A) Equal amounts of total proteins were resolved on a 4-12% Bis-Tris NuPAGE minigel (Invitrogen) and stained with EZ-Blue™(Sigma). The protein profiles of the Δ*hpf* null mutant and the WT strain were nearly identical, whereas Δ*ywlG* allele-containing strains produced a distinct proteome that was marked by six identifiable proteins. S1, ClpB; S2, DnaK; S3, GroL; S4, Zwf; S5, GudB; and S6, Fdh **(B)** Immunoblot validation of the higher DnaK levels in the Δ*ywlG* mutant than in the WT at 96 hr in TSB-glucose cultures.

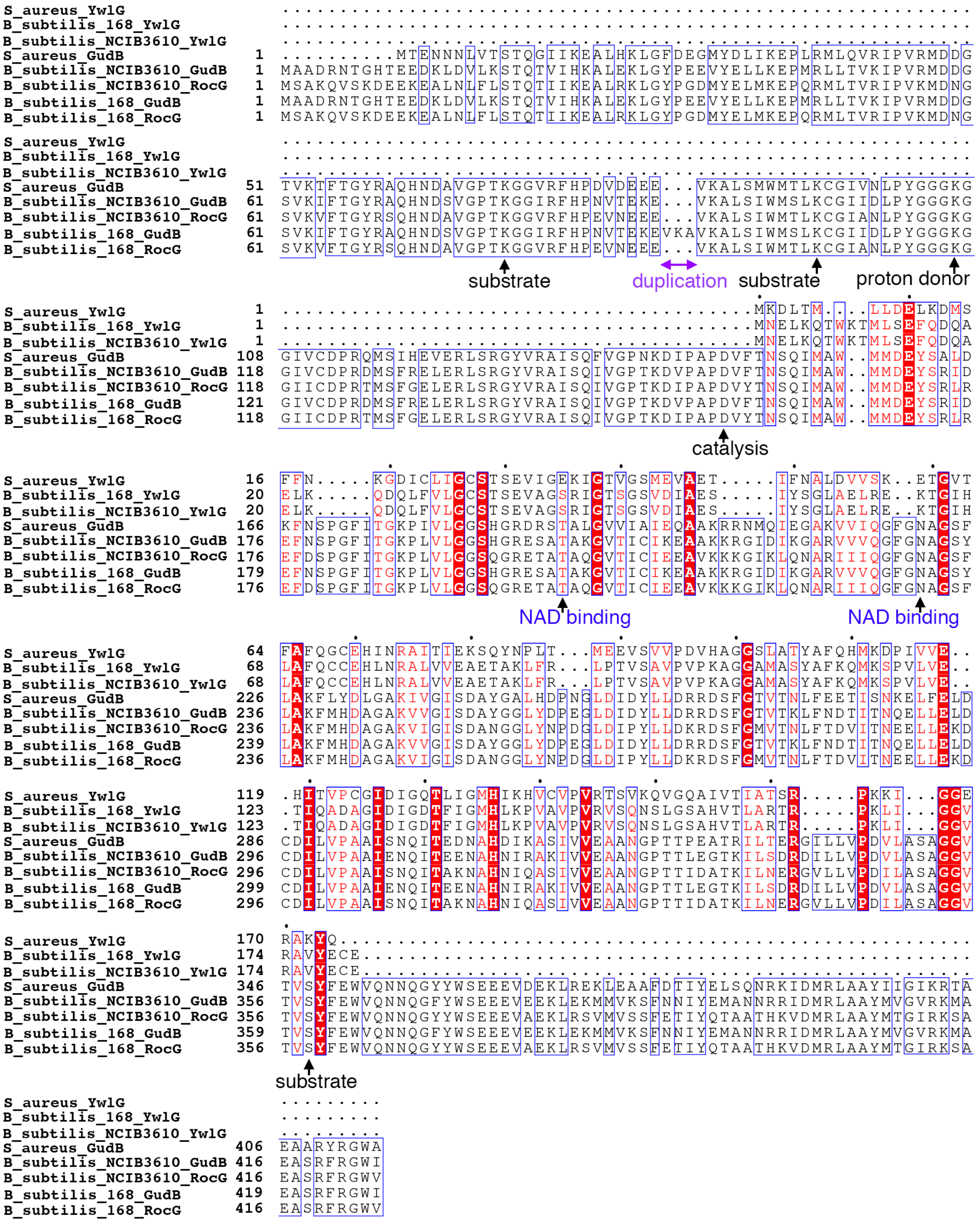

Figure S7. Multiple sequence alignment of YwlG homologs and glutamate dehydrogenases (RocG and GudB). Undomesticated *B. subtilis* NCIB1306 carries functionally active RocG and GudB enzymes. Domesticated *B. subtilis* 168 has a cryptic inactive GudB due to a 3-amino acid (VKA, purple double arrow) duplication in its active site. Substrate and co-factor binding sites, as well as catalytic sites, are indicated based on the functional assignment of NCIB1306 GudB. The accession numbers of the sequences are as follows: *S. aureus* YwlG (UniProtKB Q2FF14), *B. subtilis* NCIB1306 YwlG (GenBank AQZ925369.1), *B. subtilis* 168 YwlG (UniProtKB P39157), *S. aureus* GudB (GenBank A0A0H2XHC2), *B. subtilis* NCIB1306 RocG (GenBank AQZ926565.1), *B. subtilis* NCIB3610 GudB (GenBank AQZ91197.1), *B. subtilis* 168 RocG (UniProtKB P39633), and *B. subtilis* 168 GudB (UniProtKB P50735).

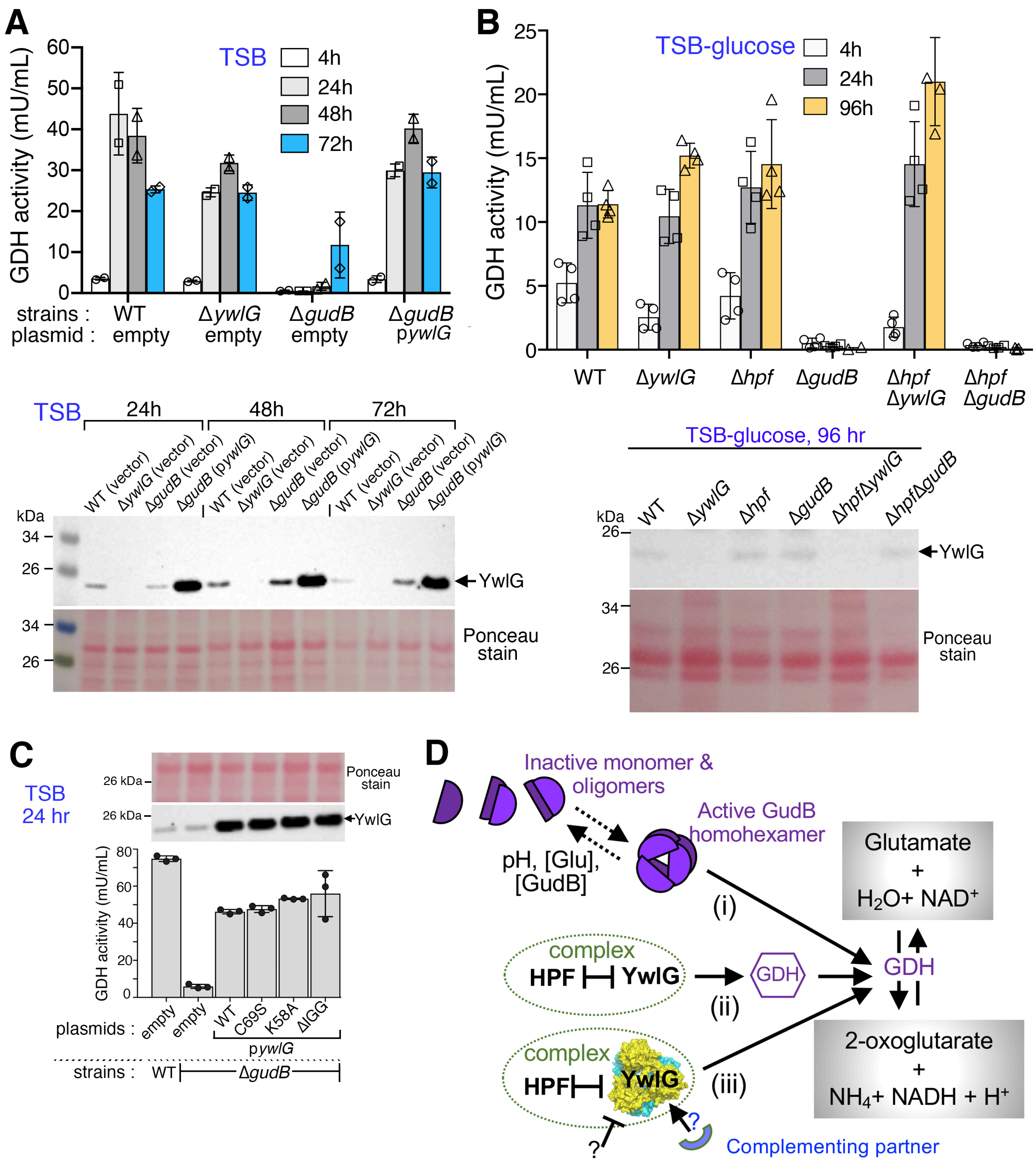

Figure S8. **Relationships of HPF, YwlG and GudB with glutamate dehydrogenase (GDH) activity. (A)** Plasmid-encoded YwlG restores GDH activity in a Δ*gudB* mutant in plain TSB medium (top). Western blot showing the expression levels of YwlG. **(B)** GDH activity in TSB-glucose medium (top). Almost no GDH was detected in the Δ*hpf*Δ*gudB* double mutant, which lacked the major provider (GudB) of GDH activity. Western blot showing the basal expression of YwlG in TSB-glucose medium (bottom). Deletion of *gudB* did not affect the YwlG level. **(C)** Overexpression of various YwlG derivatives partially restores GDH activity of Δ*gudB* mutant. **(D)** Schematic relationships among HPF, YwlG and GudB and possible roles of YwlG in GDH activity. (i) GudB is the only characterized GDH in *S. aureus*. Oligomerization of the GudB homolog is governed by intracellular pH, glutamate availability and GudB protein concentrations (15). (ii) Overexpression of YwlG bypasses HPF repression and either directly or indirectly renders GDH activity. (iii) YwlG could be an alternative GDH whose enzymatic activity is tightly regulated in part by HPF (in plain TSB medium) and an unknown factor. YwlG could also be a component of GDH complex that requires another complementation partner for full GDH activity.

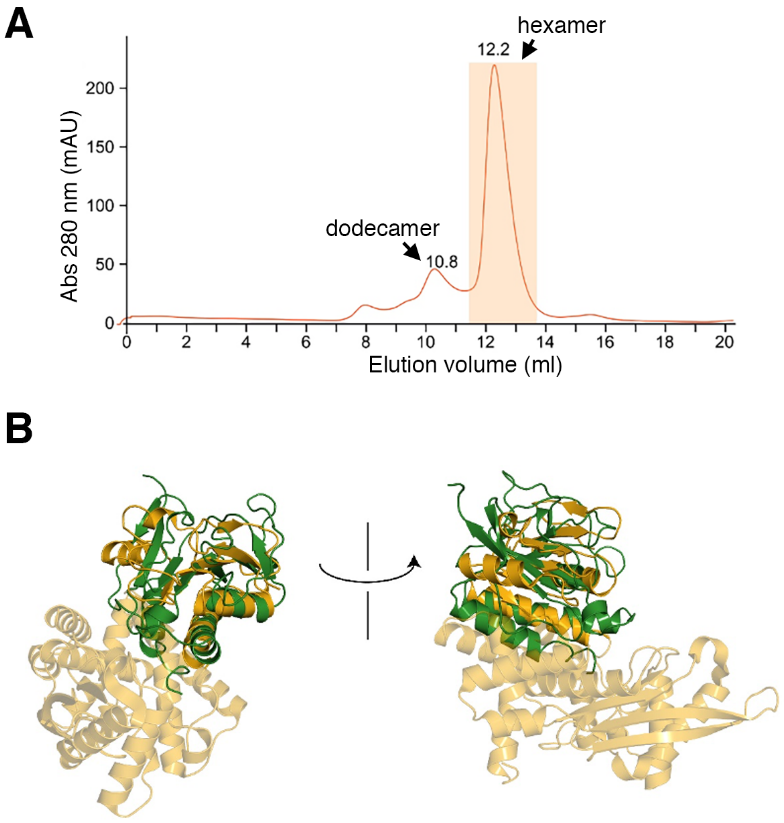

Figure S9. Analysis of *S. aureus* YwlG. **(A)** Size exclusion chromatogram of YwlG eluted from a Superdex 200 10/300 column. The shaded area indicates the peak fraction collected and used for protein crystallization. YwlG monomer: 18.9 kDa, hexamer: 113.4 kDa. **(B)** Structural superposition of *S. aureus* YwlG (green, PDB 7Z06 (this study)) and *B. subtilis* GudB1 (golden; PDB code 3K8Z). YwlG monomer can be superimposed with the C-terminal domain of GudB1 monomer with an R.M.S.D of 3.2 Å.

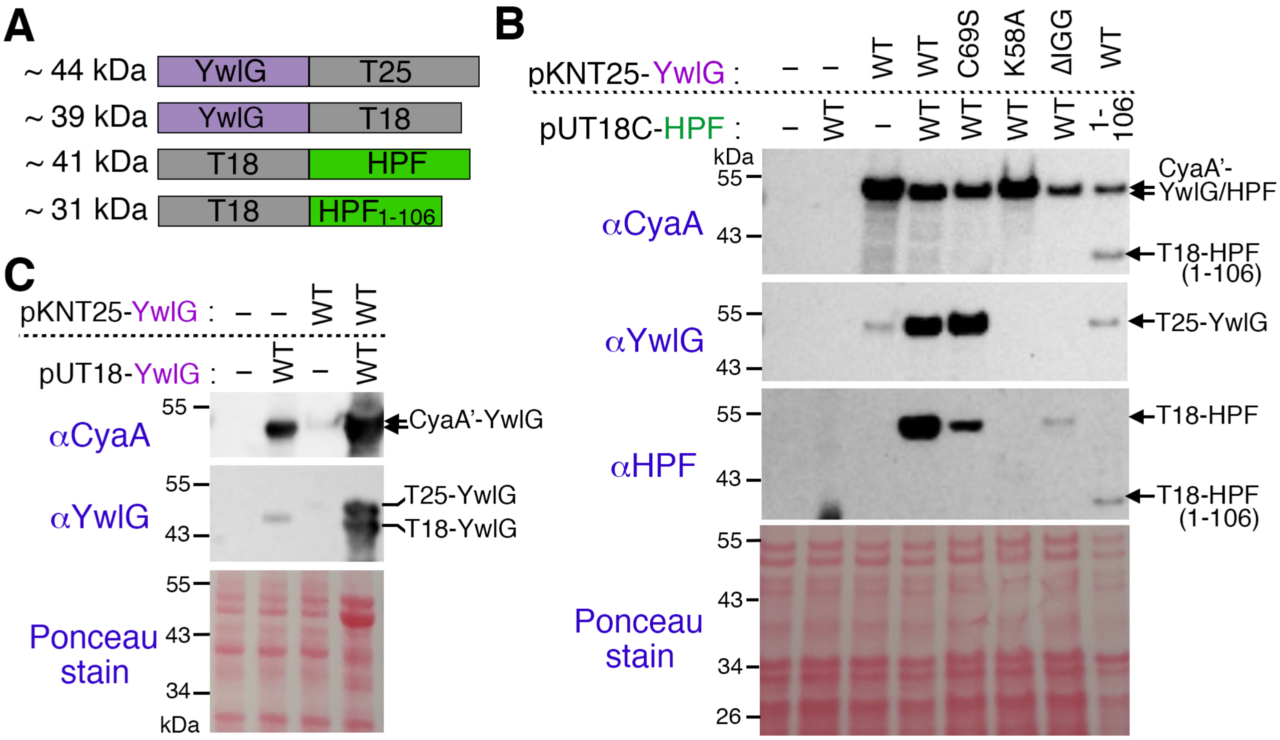

Figure S10. Expression levels of CyaA'-YwlG and CyaA'-HPF chimeras as determined by immunoblotting. (A) Diagram of CyaA' chimeric constructs and their estimated molecular weights. **(B)** Two-plasmid expression system of CyaA'-YwlG and CyaA'-HPF in *E. coli*. With the exception of YwlG(K58A) and YwlG(ΔIGG) derivatives, all other CyaA' chimeric proteins are stably expressed. **(C)** YwlG-T25 and YwlG-T18 produce similar levels of CyaA' fusion proteins when co-expressed on two separate plasmids.

Table S1. Data collection and refinement statistics.

	YwlG (UniProKB Q2FF14)
Wavelength	
Resolution range	47.99 - 1.74 (1.81 - 1.74)[a]
Space group	$P2_1$
Unit cell	54.7 136.9 79.2 90 98.6 90
Total reflections	356,927 (32,081)[a]
Unique reflections	114,645 (10,476)[a]
Multiplicity	3.1 (3.1)[a]
Completeness (%)	97.8 (88.8)[a]
Mean I/sigma(I)	10.42 (0.86)[a]
Wilson B-factor	30.34
R-merge	0.05648 (1.236)[a]
CC1/2	0.998 (0.516)[a]
CC*	1 (0.825)[a]
Reflections used in refinement	114,504 (10,390)[a]
Reflections used for R-free	5,723 (515)[a]
R-work	0.250 (0.448)[a]
R-free	0.275 (0.456)[a]
CC (work)	0.954 (0.617)[a]
CC (free)	0.948 (0.581)[a]
Number of non-hydrogen atoms	8562
macromolecules	8078
ligands	80
solvent	404
Protein residues	1058
RMS (bonds)	0.012
RMS (angles)	1.50
Ramachandran favoured (%)	96.05
Ramachandran allowed (%)	3.37
Ramachandran outliers (%)	0.58
Rotamer outliers (%)	1.33
Clash score	4.57
Average B-factor	18.86
macromolecules	17.40
ligands	55.07
solvent	40.90

[a] Statistics for the highest-resolution shell are shown in parentheses.

Table S2. Strains and plasmids.

Name	Genotypes and relevant features	Source
S. aureus		
JE2	Parental strain, plasmid cured LAC USA300 strain	BEI resources (1)
RN4220	*sau1*⁻, *hsdR*⁻, *mec*⁻, *rsbU*⁻, *agr*⁻, plasmid passage host	ATCC NR-45946
MNY133	JE2 Δ*hpf*::Km	(16)
NE838	JE2 Δ*hpf*::Erm	BEI resources
NE361	JE2 Δ*ywlG*::Erm	BEI resources
MNY250	JE2 Δ*ywlG*::Spc	This work
NE518	JE2 Δ*gudB*::Erm	BEI resources
NE976	JE2 Δ*clpB*::Erm	BEI resources
MNY252	JE2 Δ*clpB*::Spc	This study
MNY249	JE2 Δ*efp*::Km	This study
MNY236	JE2 Δ*ywlG*::Erm, Δ*hpf*::Km	This study
MNY253	JE2 Δ*ywlG*::Erm, Δ*clpB*::Spc	This study
MNY255b	JE2 Δ*hpf*::Km, Δ*gudB*::Erm	This study
E. coli		
BL21(DE3)	F⁻ *ompT gal dcm lon hsdS*$_B$($r_B^-m_B^-$) λ(DE3 [*lacI lacUV5-T7p07 ind1 sam7 nin5*]) [*malB*$^+$]$_{K-12}$(λ^S)	Lucigen
DC10B	DH10B Δ*dcm*	(17)
BTH101	F⁻, *cya-99, araD139, galE15, galK16, rpsL1 (Str*^{r}*), hsdR2, mcrA1, mcrB1.* Reporter strain for BACTH plasmids.	(18)
**Plasmids**		
pEPSA5	*E. coli-S. aureus* shuttle vector, pT5X xylose-inducible promoter, Amp^R, Cm^R	(6)
pBT2	Temperature sensitive, *E. coli-S. aureus* shuttle vector, Amp^R, Cm^R	(2)
pBTK	Temperature sensitive, *E. coli-S. aureus* shuttle vector, 1.4-kb *aph-A3* cloned into *SmaI* site of pBT2, Amp^R, Cm^R, Km^R	(3)
pDEST-Q2FF14	IPTG inducible overexpression plasmid carrying Strep-tag II fused YwlG. Km^R.	This study
pSPC	Amp^R, Spc^R, ts, marker exchange plasmid to swap the Erm-tagged transposon mutants with a spectinomycin resistance cassette.	(1)
pLI50	*E. coli-S. aureus* shuttle vector, promoterless Amp^R, Cm^R	Addgene (19)
p*ywlG*	pP$_{ywlG}$-*ywlG, ywlG* bearing its native promoter on pLI50, Amp^R, Cm^R	This study
p*ywlG* (K58A)	*ywlG*(K58A) bearing its native promoter on pLI50, Amp^R, Cm^R	This study
p*ywlG* (C69S)	*ywlG*(C69S) bearing its native promoter on pLI50, Amp^R, Cm^R	This study
p*ywlG* (ΔIGG)	*ywlG*(ΔIGG) bearing its native promoter on pLI50, Amp^R, Cm^R	This study
p*hpf*	*hpf* bearing its native promoter on pLI50, Amp^R, Cm^R	(5)

p*hpf*$^{2\times FLAG}$	2×FLAG-*hpf* bearing its native promoter on pLI50, Amp^R, Cm^R	This study
pKNT25	BACTH vector carrying T25 *cyaA (1-224)*, Km^R	(18)
pUT18C	BACTH vector carrying T18 *cyaA(225-399)*, Amp^R	(18)
p5*ywlG*	pEPSA5-YwlG, *ywlG* under the control of a xylose-inducible promoter	This study
pKNT25-YwlG	YwlG (WT)-T25 fusion on pKNT25, Km^R	This study
pUT18C-HPF	T18-HPF (WT) fusion on pUT18C, Amp^R	This study
pKNT25-YwlG(K58A)	YwlG(K58A)-T25 fusion on pKNT25, Km^R	This study
pKNT25-YwlG(C69S)	YwlG(C69S)-T25 fusion on pKNT25, Km^R	This study
pKNT25-YwlG(ΔIGG)	YwlG(ΔIGG)-T25 fusion on pKNT25, Km^R	This study
pUT18C-HPF(E107stop)	T18-HPF(1-106) fusion on pUPT18C, Amp^R	This study
pUT18-YwlG(WT)	C-terminal fusion of YwlG to T18 on pUT18, Amp^R	This study

Table S3. Primers used in this study.

Primer	Sequence (5'-3')[a]	Application
P1591(SacI)<br>P1592<br>(SmaI)<br>P1593<br>(SmaI)<br>P1594 (SalI)	AATGGAGCTCAATTTTGTGATCCACATCCAGCTA<br>TGCCGCGTACTCTGCGCCCGGGATAATCATTCAGTTTCCTCCT<br>CATTTTACAC<br>ATCCCGGGCGCAGAGTACGCGGCATAATCTCTAATTTGTTAACAAAT<br>AGCTTGTATTC<br>TTGTTGTCGACTCGCTAATTTATATGCATAAACAGT	Primers to create pBT2Δ*efp*::Km for allelic exchange
P1595<br>P1596	AGACCTTATAGTTTTAACATAAGC<br>CTTTATTAGGCGTTTTAACAGTGATTTGT	To confirm chromosomal Δ*efp*::Km allele
P1659<br>(KpnI)<br>P1660<br>(HindIII)	ATGGTACCACATTGCATGAATATGTAAAAG<br>ATAAGCTTAAAATCTCCCCTTCTTTCAAAAAAACTTA	Primers to clone *ywlG* region bearing its native promoter
P1661<br>P1662	TTAGATGTTGTGAGTGCAGAGACAGGCGTTAC<br>GTAACGCCTGTCTCTGCACTCACAACATCTAA	Quikchange primers to create YwlG(K58A)
P1663<br>P1664	TTTGCTTTTCAAGGAAGTGAACATATCAACA<br>TGTTGATATGTTCACTTCCTTGAAAAGCAAA	Quikchange primers to create YwlG(C69S)
P1710<br>P1711	ACATCTAGGCCGAAAAAGGAACGTGCTAAATACCAA<br>TTGGTATTTAGCACGTTCCTTTTTCGGCCTAGATGT	Quikchange primers to create YwlG(ΔIGG)
P1667<br>P1668	GATCGAGGAGATCAATAAGTGTTTGTTGCCG<br>CGGCAACAAACACTTATTGATCTCCTCGATC	Quikchange primers to create HPF(E107stop)
P1659<br>P1670	ATGGTACCACATTGCATGAATATGTAAAAG<br>ATAAGCTTAAAATCTCCCCTTCTTTCAAAAAAACTTA	*ywlG* and its promoter region
P1414<br><br><br>P1415	GAGCTGAAGAGCGAGACTACAAAGACGATGACGACAAGGGATC<br>AGACTACAAAGACGATGACGACAAGAACGATGATTTATACGCA<br>GGTATTGATTTAAT<br>CCTGCGTATAAATCATCGTTCTTGTCGTCATCGTCTTTGTAGT<br>CTGATCCCTTGTCGTCATCGTCTTTGTAGTCTCGCTCTTCAGC<br>TCTTAACGTAAC	Primers to introduce 2×FLAG in HPF.
P1739<br><br>P1740	GAATTCGAAAGGAGGGGCATAATGAAAGATTTGACAATGTTAT<br>TAGACGAATTAAAAG<br>CATTATGCCCCTCCTTTCGAATTC	Primers to clone *ywlG* into pEPSA5 plasmid.
P1741<br><br>P1742	GATAACAATTTCACACAGGAAACAGCTATGAAAGATTTGACAA<br>TGTTATTAGACGAATTAAAAG<br>CATGCAAGCTTGGCGTAATCATGGTTTATTGGTATTTAGCACG<br>TTCGCCAC	Primers to fuse YwlG in frame to the CyaA-T25 fragment
P1743<br><br>P1744	GTACCGAGCTCGAATTCATCGATAATTAGATTTGAAATTCATG<br>GAGATAACCTCAC<br>ATTGTACTGAGAGTGCACCATATTACTTAGTTATTG<br>TTCACTAGTTTGAATCAAGCCATATTTAC	Primers to fuse HPF in frame to the CyaA-T18 fragment.

[a]: restriction enzyme cleavage sites and mutated sites are underlined

SI REFERENCES

1. Fey PD*, et al.* (2013) A genetic resource for rapid and comprehensive phenotype screening of nonessential *Staphylococcus aureus* genes. *mBio* 4(1):e00537-00512.
2. Bruckner R (1992) A series of shuttle vectors for *Bacillus subtilis* and *Escherichia coli*. *Gene* 122(1):187-192.
3. Fuller JR*, et al.* (2011) Identification of a lactate-quinone oxidoreductase in *Staphylococcus aureus* that is essential for virulence. *Front Cell Infect Microbiol* 1:19.
4. Unger T, Jacobovitch Y, Dantes A, Bernheim R, & Peleg Y (2010) Applications of the Restriction Free (RF) cloning procedure for molecular manipulations and protein expression. *J Struct Biol* 172(1):34-44.
5. Basu A & Yap MN (2016) Ribosome hibernation factor promotes Staphylococcal survival and differentially represses translation. *Nucleic Acids Res* 44(10):4881-4893.
6. Forsyth RA*, et al.* (2002) A genome-wide strategy for the identification of essential genes in *Staphylococcus aureus*. *Mol Microbiol* 43(6):1387-1400.
7. Dzyubak E & Yap MN (2016) The expression of antibiotic resistance methyltransferase correlates with mRNA stability independently of ribosome stalling. *Antimicrob Agents Chemother* 60(12):7178-7188.
8. Miller JH (1972) *Experiments in Molecular Genetics* (Cold Spring Harbor Laboratory Press, Cold Spring Harbor, NY).
9. Davis AR, Gohara DW, & Yap MN (2014) Sequence selectivity of macrolide-induced translational attenuation. *Proc Natl Acad Sci U S A* 111(43):15379-15384.
10. Basu A & Yap MN (2017) Disassembly of the *Staphylococcus aureus* hibernating 100S ribosome by an evolutionarily conserved GTPase. *Proc Natl Acad Sci U S A* 114(39):E8165-E8173.
11. Ashkenazy H*, et al.* (2016) ConSurf 2016: an improved methodology to estimate and visualize evolutionary conservation in macromolecules. *Nucleic Acids Res* 44(W1):W344-W350.
12. Letunic I & Bork P (2021) Interactive Tree Of Life (iTOL) v5: an online tool for phylogenetic tree display and annotation. *Nucleic Acids Res* 49(W1):W293-W296.
13. Sievers F*, et al.* (2011) Fast, scalable generation of high-quality protein multiple sequence alignments using Clustal Omega. *Molecular systems biology* 7:539.
14. Robert X & Gouet P (2014) Deciphering key features in protein structures with the new ENDscript server. *Nucleic Acids Res* 42(Web Server issue):W320-324.
15. Noda-Garcia L, Romero Romero ML, Longo LM, Kolodkin-Gal I, & Tawfik DS (2017) Bacilli glutamate dehydrogenases diverged via coevolution of transcription and enzyme regulation. *EMBO Rep* 18(7):1139-1149.
16. Liponska A & Yap M-NF (2021) Hibernation-promoting factor sequesters *Staphylococcus aureus* ribosomes to antagonize RNase R-mediated nucleolytic degradation. *mBio* 2(4):e00334-00321. PMCID: PMC8406268.
17. Monk IR, Shah IM, Xu M, Tan MW, & Foster TJ (2012) Transforming the untransformable: application of direct transformation to manipulate genetically *Staphylococcus aureus* and *Staphylococcus epidermidis*. *mBio* 3(2):e00277-00211.
18. Karimova G, Gauliard E, Davi M, Ouellette SP, & Ladant D (2017) Protein-Protein Interaction: Bacterial Two-Hybrid. *Methods Mol Biol* 1615:159-176.
19. Lee CY, Buranen SL, & Ye ZH (1991) Construction of single-copy integration vectors for *Staphylococcus aureus*. *Gene* 103(1):101-105.
